# Age-dependent neuroinflammation response to voluntary wheel running and Metformin treatment in the frontal cortex of ovariectomized female mice

**DOI:** 10.1038/s41598-025-10014-0

**Published:** 2025-07-21

**Authors:** Konstancja Grabowska, Mateusz Grabowski, Julia Morys, Edyta Olakowska, Andrzej Małecki, Jarosław J. Barski, Marta Nowacka-Chmielewska

**Affiliations:** 1 Department of Physiology, Faculty of Medical Sciences in Katowice, Medical University of Silesia, Katowice, Poland; 2 Laboratory of Molecular Biology, Institute of Physiotherapy and Health Sciences, Academy of Physical Education, Katowice, Poland

**Keywords:** Neuroinflammation, NLRP3 inflammasome, Voluntary wheel running, Metformin, Ovariectomy, Physical activity, Neuroimmunology, Menopause, Neurophysiology

## Abstract

In postmenopausal women, estrogen deficiency can exacerbate inflammation associated with aging, increasing the risk of neuroinflammatory disorders. Although physical activity (PA) may reduce this risk, it may not be feasible for all patients. Metformin, an anti-diabetic drug, has been proposed as an exercise mimetic due to its ability to reduce systemic inflammation. We investigated whether the voluntary wheel running (VWR) or metformin treatment (MF) impacts neuroinflammation of female mice following ovariectomy. Middle-aged (A) and young adult (Y) mice underwent ovariectomy (OVX) or sham operation (SHAM), and had access to VWR or MF in their drinking water for six weeks. A sedentary control group received water without metformin. The frontal cortex and serum samples were collected for further evaluation using western blot, RT-qPCR, and ELISA methods. Ovariectomy reduced running activity in both age groups. A decrease in NLRP3 expression was observed in middle-aged mice following PA. The NF-κB p-p65/p65 ratio increased in OVX/MF_Y mice compared to SHAM/MF_Y mice. Our results indicate a modulatory effect of running and metformin on neuroinflammation with varying effects that seem age-related and ovarian status-dependent. The responsiveness to PA and MF in the frontal cortex of adult mice could be mainly associated with the NF-κB p65 signal.

**Supplementary Information:**

The online version contains supplementary material available at 10.1038/s41598-025-10014-0.

## Introduction

The life expectancy of both women and men has considerably increased in recent years. Despite the rise in women’s life expectancy, the average age of menopause remains relatively unchanged, leading to a more extended postmenopausal period. Most women experience menopausal symptoms, which are more prevalent during perimenopause and postmenopause. While some menopausal symptoms, such as vasomotor symptoms, may alleviate over time, others, like genitourinary symptoms, tend to worsen, which consequently may reduce the quality of life. Additionally, epidemiological studies indicate that women are more vulnerable than men to developing central nervous system (CNS)-related disorders, which may be linked to the adverse effects of estrogen deficiency^1–3^. Estrogens play a critical role in developing and regulating the reproductive system and menstrual cycle in females but also exert diverse non-reproductive actions^4^. Clinical^5–7^ and experimental^8–13^ studies have reported neuroprotective effects of estrogens in several CNS disorders, including multiple sclerosis (MS), Parkinson’s disease (PD), Alzheimer’s disease (AD), spinal cord injury, and stroke. The regulation of neuroinflammation alongside synaptic plasticity and hippocampal neurogenesis enhancement has been suggested as some of the key processes underlying the neuroprotective effect of estrogens^14,15^. While neuroinflammation can play a beneficial role in the development of the CNS, memory, learning, neuroprotection, and tissue repair, negative consequences may arise when the inflammatory stimulus persists. This may lead to prolonged or maladaptive neuroinflammation responses, which is a significant contributing factor to many neurological diseases^16,17^. Additionally, estrogen deficiency may exacerbate the central and peripheral inflammation that occurs during normal aging, thereby increasing the risk of developing brain-inflammation-related disorders. Menopause has been associated with changes in the expression of genes regulating the inflammatory response within the forebrain^18^. This alteration may increase the risk of developing neuroinflammation-related CNS disorders^19^. Neuroinflammation is mediated by intracellular multiprotein complexes, known as inflammasomes. The activation of a nucleotide-binding oligomerization domain-like receptor (NLR) family pyrin domain containing 3 (NLRP3) inflammasome promotes caspase 1-dependent secretion of proinflammatory cytokines, interleukin (IL)−1β, and IL-18, and has been linked with AD and PD development^20,21^.

Introducing lifestyle changes, such as physical activity (PA), may reduce the risk of developing CNS disorders in postmenopausal women^22^. In elderly women, PA reduces systemic inflammation and maintains concentrations of peripheral markers for blood-brain barrier (BBB) integrity^23^. Also, PA protects from cognitive deficits by influencing neurotrophin and neurotransmitter levels, enhancing synaptic plasticity, and preventing hippocampal atrophy. In early postmenopausal women, regular moderate-intensity PA supports cognitive functions and reduces the risk of neurodegenerative diseases^24–28^. To simulate human endurance training in rodents and evaluate the effects of PA on physiological processes, treadmill exercise or voluntary wheel running could be applied. Unlike other experimental exercise models in mice, wheel running is a voluntary activity that does not require additional external stimulus or enforcement^29^. Experimental studies indicate that treadmill exercise and voluntary wheel running may contribute to improving the course of neurodegenerative diseases^30–32^, stroke^33,34^, metabolic disorders^35,36^, inflammation^37,38^, and aging^39,40^, among others, through the positive effect on neuroinflammation processes.

However, PA cannot be applied as a therapeutic approach for all patients, particularly those with physical, intellectual, or visual disabilities. Many individuals with disabilities do not meet the World Health Organisation’s PA guidelines. Additionally, in middle-aged and elderly populations, factors such as socioeconomic status, lack of motivation, and health-related issues are significant barriers to PA^41–43^. Thus, to eliminate the effects of PA lack, pharmacological intervention may be considered and involve substances capable of activating cellular mechanisms similar to those observed after regular exercises, such as exercise mimetics (EMs)^44^. Metformin, well known antidiabetic drug, was previously documented that may inhibit gluconeogenesis and lipogenesis while promoting fatty acid oxidation and improving insulin sensitivity. Consequently, metformin may regulate carbohydrate metabolism, supports weight loss, and reduces the risk of developing type 2 diabetes^45^. Among women, metformin is widely used in the treatment of metabolic disorders as well as reduces the risk of developing breast cancer^46,47^. Metformin has anti-inflammatory effects and improves insulin sensitivity in patients with polycystic ovary syndrome^48^. Therefore, in this study, we proposed that metformin may be a suitable EM to address neuroinflammatory processes which was supported by evidence showing that metformin reduces systemic inflammation^49,50^, and might, in turn, improve cognitive functions^51,52^. Additionally, metformin treatment may lower the risk of AD^53^ and prevent brain atrophy among the elderly^54^. The increasing evidence from animal studies indicates that the neuroinflammation modulatory effects of metformin use may be linked to the activation of AMP-activated protein kinase (AMPK)^55,56^. It might inhibit the NF-κB signaling pathway and NLRP3 inflammasome activation, reduce oxidative stress, and improve BBB integrity^55–57^. The molecular mechanisms underlying neuroinflammation in postmenopausal women remain unclear. Therefore, to model the changes related to postmenopause in the CNS, we used the established rodent menopause model - ovariectomy. Since postmenopause occurs with aging while ovariectomy represents a sudden cessation of ovarian function, we utilized a middle-aged mouse that may experience reproductive senescence^58^. Thus, the present study aimed to characterize the effect of voluntary wheel running and metformin treatment on neuroinflammatory changes related to ovariectomy in middle-aged female mice, focusing on the modulation of the NLRP3 inflammasome. This study describes the changes in the frontal cortical protein and gene expression levels involved in NLRP3 inflammasome activation and NF-κB signaling pathway. The findings open up a discussion about the role of voluntary PA and metformin as a strategy for modulating neuroinflammatory processes. Moreover, changes in neuroinflammation and low-grade, chronic, systemic inflammation can occur during aging, regardless of estrogenic state^58,59^. Therefore, we decided to conduct our study on middle-aged and young adult female mice to explore differences in age-related neuroinflammatory responses to PA and metformin.

## Methods

### Animals

The nulliparous female C57BL/6 N mice were provided by the Animal House of the Department for Experimental Medicine (Medical University of Silesia, Katowice, Poland). Animals were treated using the protocols approved and monitored by the Local Committee for the Care and Use of Laboratory Animals in Katowice, Poland (permission no. 61/2020, 30th November 2020, permission no. 18/2023, 31 st March 2023), and all experiments and methods were performed in accordance with relevant guidelines and regulations. Before entering the experiment, mice were housed 5–8 per cage. The minimum number of mice required to obtain consistent data was used, and every effort was taken to minimize the suffering of the animals. Mice were housed in a climate-controlled room (22 ± 2 °C, humidity: 40–60%) with a 12 h:12 h light/dark cycle starting at 07:00 a.m. with food and water *ad libitum*.

### Experimental design

#### Experiment 1

**Fig. 1:**
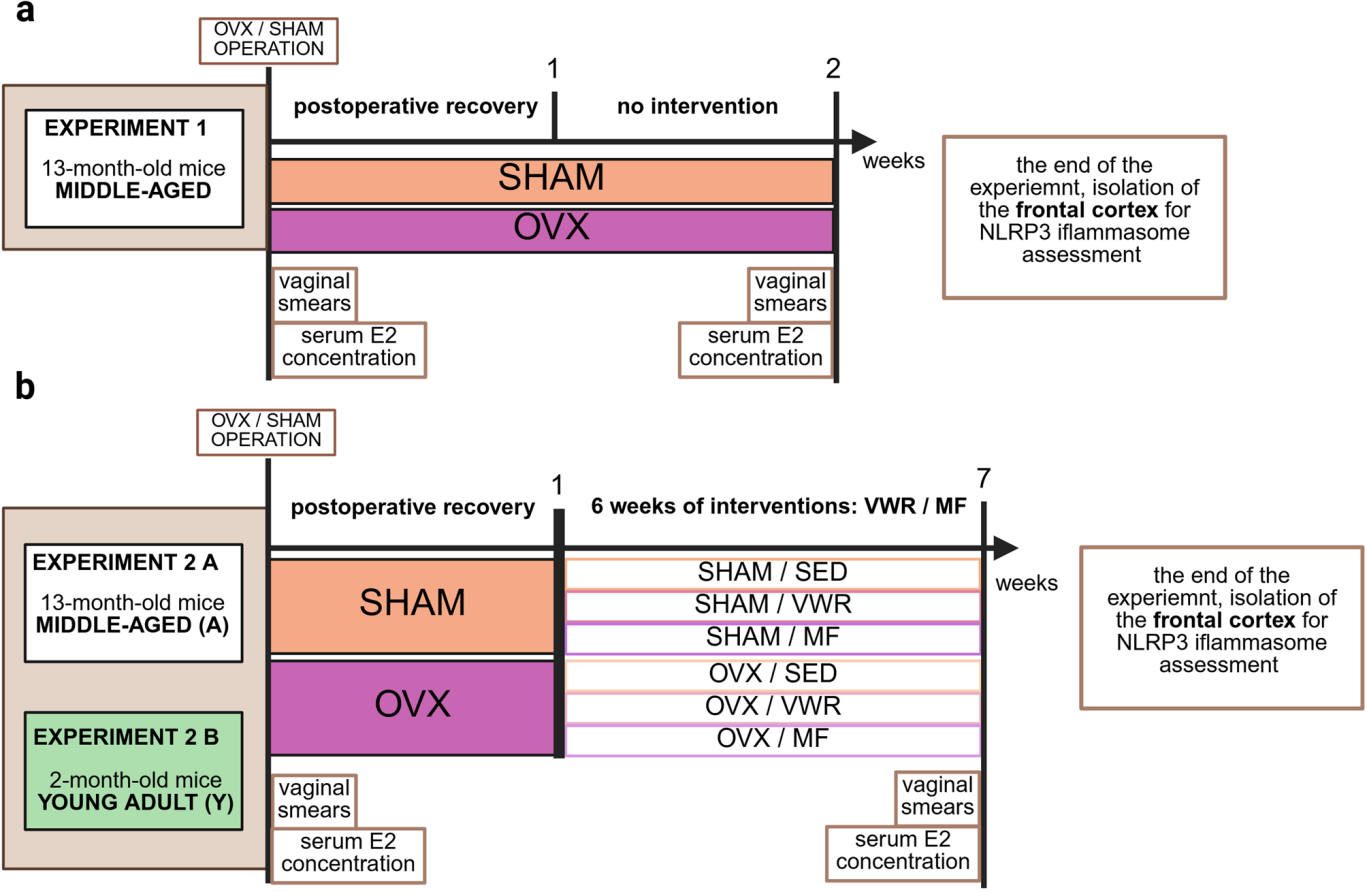
Schemes of the conducted experiment. Evaluation of the effects of short-term (two weeks) ovariectomy in middle-aged mice (**a**) and long-term (seven weeks) ovariectomy with voluntary wheel running (VWR) or metformin (MF) treatment in middle-aged (A) (**b**) and young adult (Y) mice (**b**) on the neuroinflammatory responses in the frontal cortex. SHAM - sham operation, OVX - bilateral ovariectomy, SED - sedentary, E2–17-β-estradiol. Created in BioRender.com.

The middle-aged (13-month-old) (*n* = 10) (~ 46.56–47.71 years old human^60^), female mice were randomly divided into two groups. One group underwent a bilateral ovariectomy (OVX, *n* = 5), while the control group received a sham operation (SHAM, *n* = 5), which simulated an ovariectomy without removing both ovaries. Following the operations, the mice were housed without intervention for the next two weeks, which included one week of postoperative recovery (Fig. 1a).

#### Experiment 2 A

The middle-aged (13-month-old, A) (*n* = 46) (~ 46.56–50 years old human^60^), female mice were randomly divided into two groups. In one group, animals were bilaterally ovariectomized (OVX, *n* = 23), while in the other group, mice underwent a sham operation (SHAM, *n* = 23). After operations, animals were housed individually for one week of postoperative recovery without any interventions. Next, ovariectomized and sham-operated animals were divided into six experimental groups in a body mass-matched manner. Over the next six weeks, the mice were introduced to one of two interventions. One cohort of mice was subjected to voluntary wheel running (OVX/VWR_A, SHAM/VWR_A, *n* = 8 per group). At the same time, animals in the OVX/MF_A (*n* = 6) and SHAM/MF_A (*n* = 6) groups were treated with metformin given in drinking water. The control groups, consisting of sedentary ovariectomized (OVX/SED_A, *n* = 9) and sham-operated (SHAM/SED_A, *n* = 9) animals, had free access to drinking water and were housed without any intervention during the six weeks (Fig. 1b).

#### Experiment 2 B

The young adult (2-month-old, Y) (*n* = 27) (~ 15–22.8 years old human^60^), female mice were either bilaterally ovariectomized (*n* = 13) or sham-operated (*n* = 14). After a week, the mice were divided into six experimental groups (*n* = 4–5 per group) in a body mass-matched manner. The animals proceeded to the same experimental paradigm as in Exp. 2 A. The OVX/VWR_Y (*n* = 4) and SHAM/VWR_Y (*n* = 4) groups were subjected to voluntary wheel running for six weeks. Animals receive metformin solution with drinking water in the OVX/MF_Y (*n* = 5) and SHAM/MF_Y (*n* = 5) groups. The control animals (OVX/SED_Y, *n* = 4, SHAM/SED_Y, *n* = 5) were housed without intervention (Fig. 1b).

During the experiment, mice were housed individually, with one mouse per cage. All animals had free access to standard chow (Labofeed B, Wytwórnia Pasz Morawski, see Supplementary Table 1, Additional file 1) *ad libitum* during the experiment. The estrous cycle phase assessment and 17-β-estradiol (E2) concentration measurements were performed twice: at the beginning and directly before euthanasia. At the end of the experiment, mice were anesthetized and then decapitated. The frontal cortex and blood serum samples were collected for further measurements.

### Surgical procedures

All surgical procedures were performed between 8:00 a.m. − 4:00 p.m. under aseptic conditions. Before operations, mice were fasted for 12 h. Bilateral ovariectomy was performed as described by Souza et al. (2019)^61^. In brief, mice were anesthetized (IP injection: ketamine 100 mg/kg/b.m., xylazine 10 mg/kg/b.m.) and placed on a warm electric blanket. The right flank was shaved and disinfected. Dorsolateral incisions of skin and muscle layers were performed, the right horn of the uterus was exposed, and the ovary was carefully removed, leaving the uterus intact. The procedure was applied on the second flank. Mice in the control group underwent a sham operation under the same conditions, but their ovaries remained intact. After the operations, mice were housed individually for one week to recover without any procedures. For three consecutive days post-operation, the animals were administered paracetamol (Pedicetamol, SEQUOIA SP. Z O.O.) with drinking water (24 mg/kg/b.m./day) and gentamicin (IP injection 80 mg/kg/b.m./day) (Gentamycin Krka^®^, Krka). All animals were monitored for wound healing and pain behavior.

### Estrous cycle evaluation

The estrous cycle was assessed twice during the experiment to establish the cycle phase on the days of operation and euthanasia, directly after anesthesia^62^. Vaginal lavages were conducted by gently aspirating saline into the opening of the vaginal canal. The vaginal smears were stained with Giemsa stain and subsequently examined under light microscopy to identify cell types characteristic of the estrus cycle.

### Voluntary wheel running

A cohort of female mice that were either bilaterally ovariectomized (OVX/VWR_A, OVX/VWR_Y) or sham-operated (SHAM/VWR_A, SHAM/VWR_Y) was subjected to voluntary wheel running. At the beginning of the second week post-operations, each mouse from the SHAM/VWR and OVX/VWR groups was placed separately in cages equipped with integrated running wheels (ACTI-PT2-MCR2, Actimetrics). Mice had free access to the running wheel for 24 h daily over six weeks, including one week of adaptation. In the remaining sedentary and metformin-treated groups, the animals were housed in the same manner as running mice, but their running wheels were immobilized. The distance covered by each mouse was recorded individually using *ClockLab Data Collection* and analyzed with *ClockLab Analysis* (Actimetrics).

### Metformin administration

A cohort of bilaterally ovariectomized (OVX/MF_A, OVX/MF_Y) and sham-operated (SHAM/MF_A, SHAM/MF_Y) female mice were treated with metformin (metformin hydrochloride, M0605000, Sigma-Aldrich). From the second week, the mice had free access to drinking water with metformin solution *ad libitum* for six weeks. As controls, sedentary or running animals had access to drinking water *ad libitum*. The concentration of metformin in the drinking water was selected to achieve a daily dose of 100 mg/kg/b.m. This dosage is equivalent to a daily intake of ~ 500 mg metformin for a 60 kg person^63^. The consumption of metformin was monitored three times a week. Due to the stability in aqueous solution, the metformin was available for animal consumption for up to 72 h^64,65^.

### Serum 17-β-estradiol concentration measurement

E2 concentration was measured twice in all animals’ blood serum collected from the tip of the tail. Blood samples were collected between 8:00 a.m. and 9:00 a.m., immediately before the operation and euthanasia. The blood samples were centrifuged (2000 x g, 4 °C, 10 min), and the serum was stored at −80 °C. The concentration of E2 was measured using a commercially available ELISA kit (ab108667, Abcam) according to the manufacturer’s protocol.

### Euthanasia and tissue collection

Brain sample collection began at 9:00 a.m. Mice were anesthetized (IP injection: ketamine 100 mg/kg/b.m., xylazine 10 mg/kg/b.m.) and euthanized by decapitation. Then, the brain was rapidly removed and placed in Alto Stainless Steel 1 mm Mouse Brain Matrix (Coronal 40-75gm, Roboz Surgical Instrument Co.), dorsal side up. A brain slice was removed in the (+ 4) - (+ 2) anterior/posterior to bregma coordinates, and the frontal cortex was isolated. The collected area corresponds to the frontal association cortex (FrA), the dorsolateral part of the prelimbic area (PrL), and the motor cortex (M1, M2)^66^. The frontal cortex samples from the right hemisphere (for protein isolation) and the left hemisphere (for RNA isolation) were stored at − 80 °C until further analysis. Retro-orbital blood was collected and centrifuged at 4000 × g for 20 min (4 °C). The collected serum was stored at − 80 °C for subsequent analysis. The uterus was isolated from all animals and weighed. In the case of sham-operated animals, the uteri were weighed after the removal of the ovaries.

### Western blotting

For protein extraction, frontal cortex samples were sonicated in a RIPA buffer (20–188, Merck Millipore) with protease (04693116001, Roche Holding AG) and phosphatase (4906845001, Roche Holding AG) inhibitors and then centrifuged (12 000 x g, 20 min, 4 °C). The protein concentration was measured with Pierce BCA Protein Assay Kit (23227, Thermo Fisher Scientific) according to the manufacturer’s protocol. The samples containing 20 µg of total protein were separated on precast polyacrylamide gel (4568086, Bio-Rad Laboratories, Inc.) and transferred onto PVDF membranes (1620177, Bio-Rad Laboratories, Inc.) using. Trans-Blot^®^ Turbo™ Transfer System (Bio-Rad Laboratories, Inc.). Membranes were blocked (1 h, RT) in casein (B6429, Sigma-Aldrich) or 5% bovine serum albumin (A9418, Sigma-Aldrich) solutions and then, incubated in the blocking solution with the appropriate primary antibody (see Supplementary Table 2, Additional file 1) (overnight, 4 °C). After washing in TBST (524750, Merck Millipore, P9416, Sigma-Aldrich), the membranes were incubated with a secondary antibody (170–6515, Bio-Rad Laboratories, Inc.) (1 h, RT). After incubation with Clarity™ Western ECL Substrate (1705060, Bio-Rad Laboratories, Inc.), chemiluminescent protein detection was performed using ChemiDoc-Touch Imaging System (Bio-Rad Laboratories, Inc.). Original full-length gels and blots were included in the Additional file 2. Visualized band intensity was calculated by ImageLab Software 5.2.1 (Bio-Rad Laboratories, Inc.). According to Stain-Free Imaging Technology (Bio-Rad Laboratories, Inc.) protein band normalization was performed concerning total lane protein visualized under UV light on polyacrylamide gel after electrophoresis. The data were presented as a mean ± SD of fold change of the samples from the studied group compared to the control group.

### Evaluation of cytokine concentration

The frontal cortex and serum concentrations of IL-1β were assessed using commercially available ELISA kits according to the manufacturer’s protocol (ab197742, Abcam). The blood serum concentration of IL-18 and TNF-alpha (TNF-α) were measured using a commercially available ELISA kit (ab216165, Abcam, EM0183, Wuhan Fine Biotech Co.).

### Real-Time PCR

Total RNA was isolated using miRNeasy Mini Kit (217004, Qiagen) according to the manufacturer’s instructions. Total RNA (400 ng) was reverse transcribed with the Maxima H Minus First Strand cDNA Synthesis Kit, with dsDNase (K1682, Thermo Fisher Scientific). qPCR was performed using the Fast SG qPCR Master Mix (2x) kit (E0411, Eurx) and target-specific primers (see Supplementary Table 3, Additional file 1) in the LightCycler 96 (Roche). *Hprt1* and *Gapdh* were used as endogenous controls. The normalized relative gene expression was calculated by the 2^−ΔΔCt^ method^67^.

### Statistical analysis

Prism 10.1.2 (GraphPad Software) was used for statistical analyses and figure generation. The results are shown as the means ± standard deviation (SD). The distribution of each dataset was checked for normality using the Shapiro–Wilk test. Depending on the data distribution, the t-test with Welch correction or Mann-Whitney test was used to estimate significant differences between the two groups (western blotting, qPCR, ELISA measurements, uterus mass, characteristics of voluntary wheel running). Two-way ANOVA with Tukey’s multiple comparison tests was used to analyze data obtained from six experimental groups (Western blotting, qPCR, and ELISA measurements). Also, Two-way RM ANOVA with Šídák’s multiple comparisons test for data obtained at different time points was performed. In all analyses, p-values of less than 0.05 were considered statistically significant. The statistical analysis results were presented in the Additional file 3.

## Results

### Short-term ovariectomy affects the expression of NLRP3 inflammasome components in the frontal cortex of middle-aged mice

**Fig. 2:**
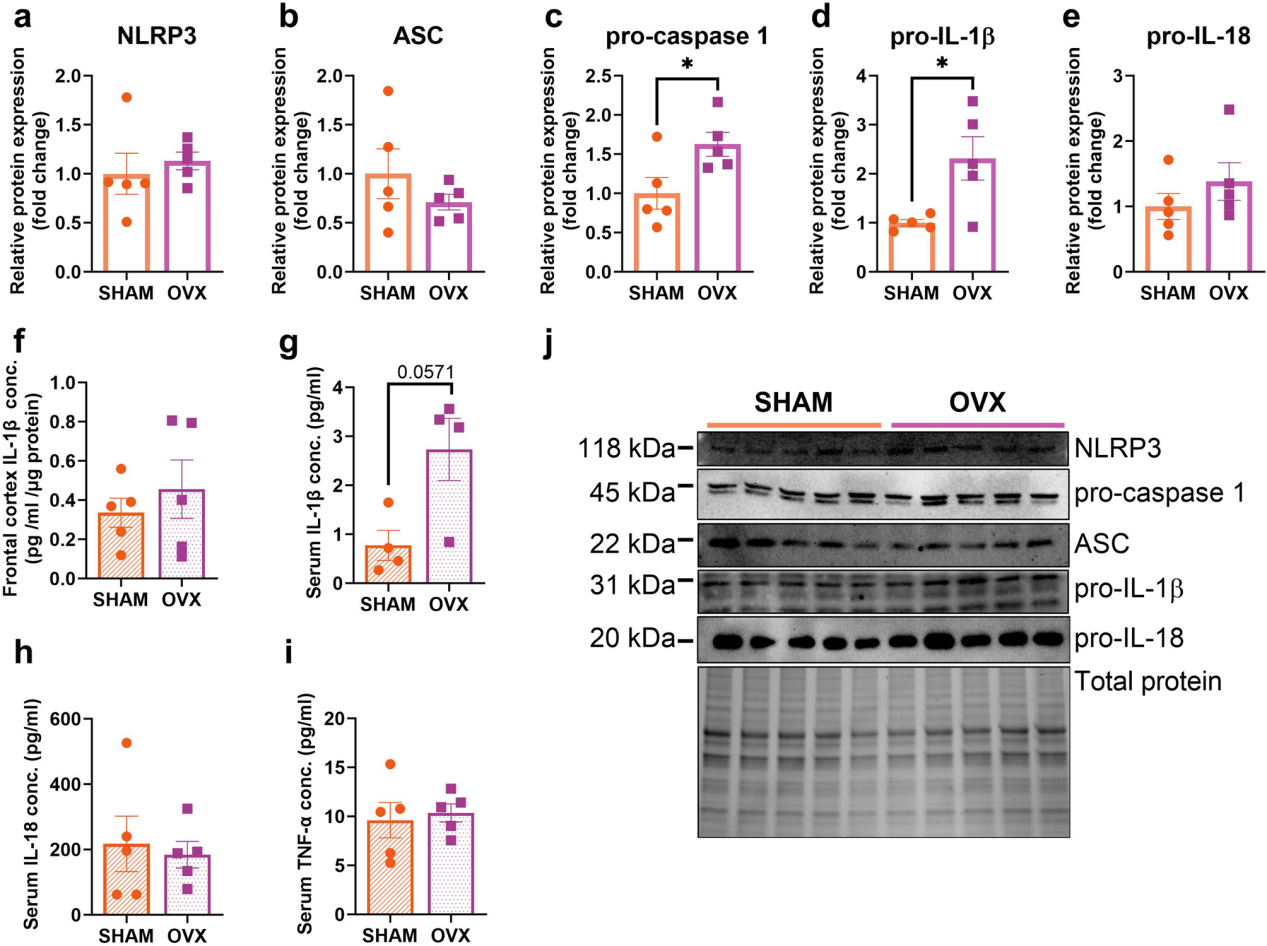
The NLRP3 inflammasome components expression level in the frontal cortex of middle-aged female mice two weeks after ovariectomy (**a**-**e**). Short-term ovariectomy (2 weeks) significantly increased pro-caspase 1 and pro-IL-1β protein expression levels (**c**, **d**). No statistically significant changes were noted in the frontal cortex (**f**) and serum IL-1β (**g**) concentration, as well as in serum IL-18 (**h**) and TNF-α (**i**) concentrations. Representative images of Western blotting analysis (**j**). Values are ​​presented as mean ± SD. Unpaired t-test with Welch’s correction (NLRP3, ASC, pro-caspase 1, pro-IL-1β, pro-IL-18, frontal cortex IL-1β conc., serum IL-18 conc., serum TNF-α conc.) and Mann-Whitney test (serum IL-1β) were performed. *p < 0.05. SHAM - sham operation, OVX - bilateral ovariectomy. *n* = 5 per group.

Short-term ovariectomy significantly increased the cortical expression of pro-caspase 1 (*p* = 0.0408) and pro-IL-1β (*p* = 0.0405) in comparison to sham-operated animals (Fig. 2c, d,j). No differences were noted in the expression levels of the NLRP3, apoptosis-associated speck-like protein containing a CARD (ASC), and pro-IL-18 proteins (Fig. 2a, b,e) or in the mRNA expression levels of *Nlrp3*, *Casp1*, *Il-1b*, and *Il-18* two weeks after operations (see Supplementary Table 4, Additional file 1).

We observed an increase in the cortical expression of pro-IL-1β in OVX animals compared to the SHAM group. Therefore, the total concentration of IL-1β in cortical homogenates was additionally assessed. Two weeks after the operations, no changes in IL-1β concentration were noted between the two experimental groups (Fig. 2f). In sera of ovariectomized mice, the concentration of IL-1β tended to increase (*p* = 0.0571) (Fig. 2g). There were no changes in the serum level of IL-18 (Fig. 2h) and TNF-α between the experimental groups (Fig. 2i).

### Short-term ovariectomy elevates the TLR4 expression but does not influence the NF-κB p65 signaling pathway in middle-aged mice

**Fig. 3:**
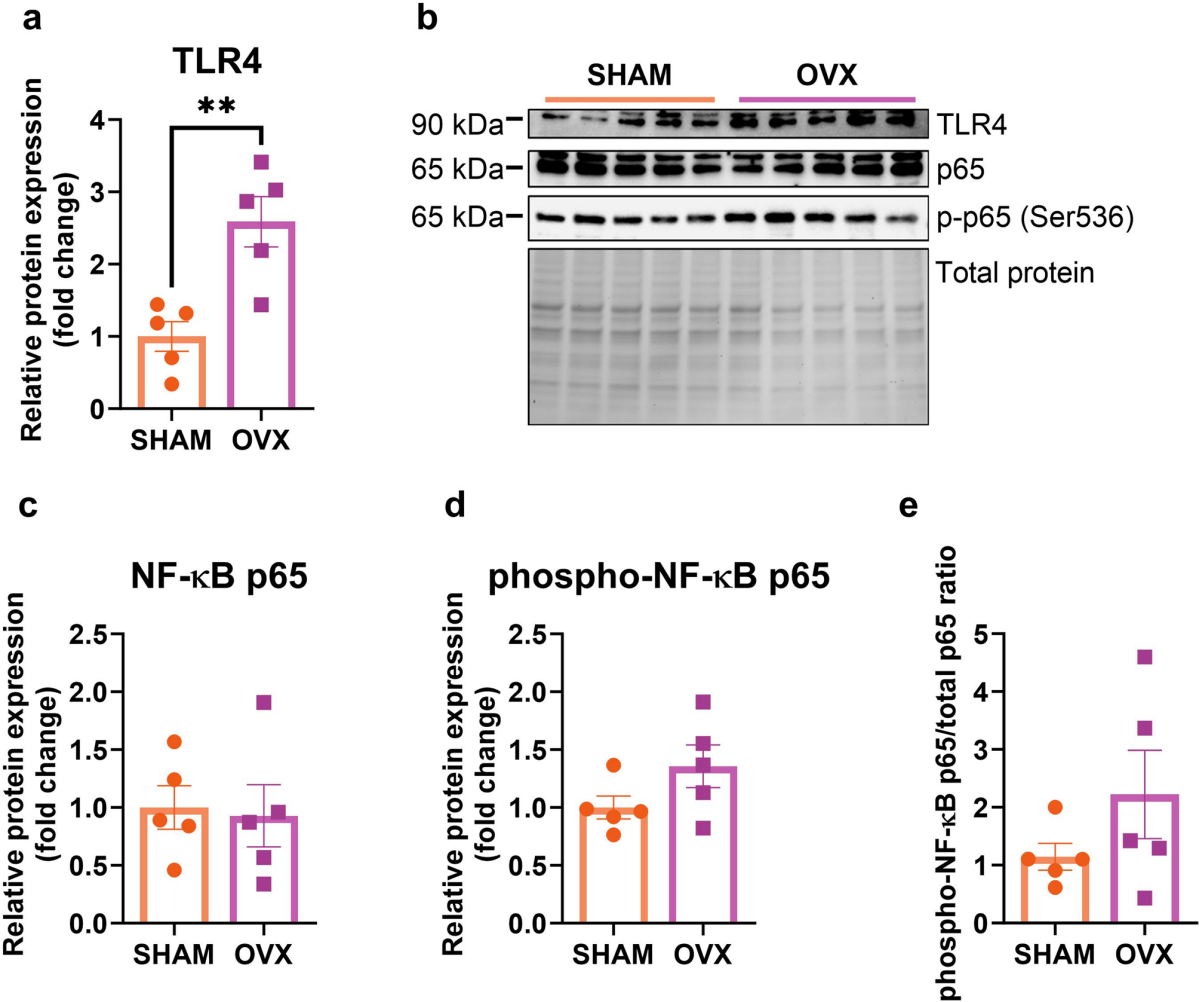
Two weeks after the operation, ovariectomized middle-aged mice had upregulated TLR4 receptor expression levels in comparison to sham-operated animals (**a**). No changes were observed in the expression of NF-κB p65 between ovariectomized (OVX) and the control animals (SHAM) (**c**-**e**). Representative images of Western blotting analysis (**b**). Values are ​​presented as mean ± SD. An unpaired t-test with Welch’s correction was performed. **p < 0.01. SHAM - sham operation, OVX - bilateral ovariectomy. *n* = 5 per group.

Ovariectomy significantly increased the toll-like receptor 4 (TLR4) expression (*p* = 0.0067) (Fig. 3a, b). No changes in the expression of the NF-κB p65 and phosphorylated p65 (Ser536) levels were observed (Fig. 3c, d). Neither the ratio of phospho-NF-κB p65 to total p65 was changed (Fig. 3e). There were no significant differences in the expression of *Tlr4*, *Rela*, *Relb*, *Nfkbia*, *Nfkbib*, and *Ikbkb* genes between the two experimental groups (see Supplementary Table 4, Additional file 1).

### Ovariectomy decreases the voluntary wheel-running activity in middle-aged and young adult mice

**Fig. 4:**
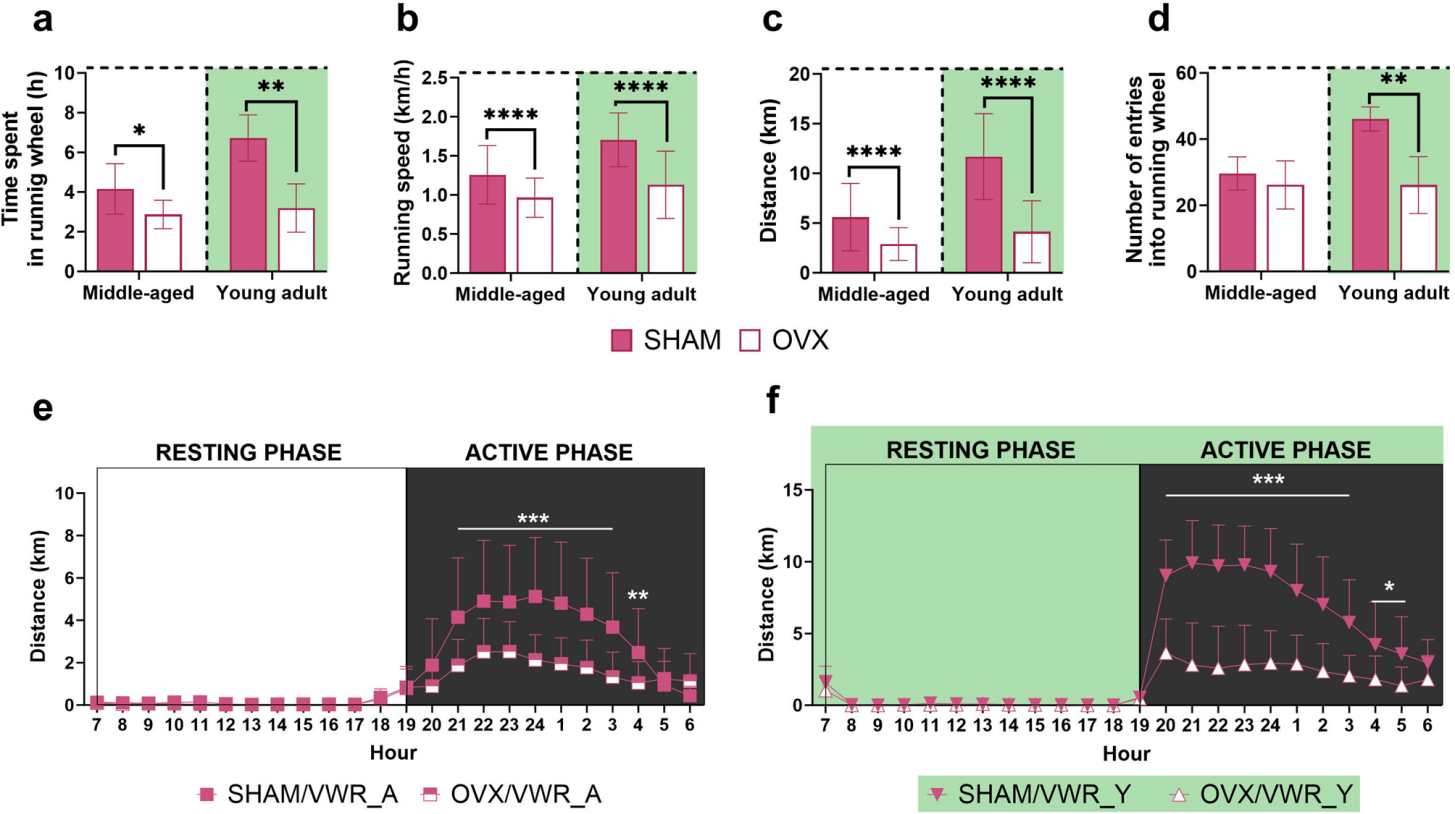
Ovariectomy reduced voluntary wheel running activity. Middle-aged and young adult female mice after ovariectomy spent less time in the running wheel (**a**), ran at lower speed (**b**), and traveled shorter distances (**c**) than sham-operated aged-matched controls. Ovariectomized young adults, but not middle-aged mice, had fewer entries into the running wheel (**d**). Middle-aged (**e**) and young adult (**f**) mice ran with the greatest intensity at night during the active phase. Values ​​are presented as mean ± SD. Unpaired t-test with Welch’s correction: Time spent in the running wheel (SHAM/VWR_A vs. OVX/VWR_A, SHAM/VWR_Y vs. OVX/VWR_Y), Number of entries into the running wheel (SHAM/VWR_Y vs. OVX/VWR_Y). Mann-Whitney test: Running speed, Distance (SHAM/VWR_A vs. OVX/VWR_A, SHAM/VWR_Y vs. OVX/VWR_Y), Number of entries into the running wheel (SHAM/VWR_A vs. OVX/VWR_A). Two-way RM ANOVA followed by Šídák’s multiple comparisons test (distance over 24 h). *p < 0.05, **p < 0.01, ****p < 0.0001. Middle-aged, A: *n* = 6–7 per group, Young adult, Y: *n* = 4 per group. SHAM - sham operation, OVX - bilateral ovariectomy, VWR - voluntary wheel running.

The middle-aged ovariectomized mice spent less time on running wheels (*p* = 0.0463) and covered shorter distances (*p* < 0.0001) than animals after the sham operation. Furthermore, animals in the SHAM/VWR_A group ran significantly faster than those in the OVX/VWR_A group (*p* = 0.0477). The frequency of entries into running wheels did not differ between groups (Fig. 4a-d). However, ovariectomy in middle-aged mice decreased running activity during the active phase. The animals in the SHAM/VWR_A group covered more than twice the distance at night, from 9:00 p.m. to 4:00 a.m. (9:00 p.m. (*p* = 0.0003), 10:00 p.m. (*p* = 0.0004), 23:00 (*p* = 0.0001), 00:00–03:00 (*p* < 0.0001), 04:00 (*p* = 0.0039)), compared to the animals in the OVX/VWR_A group (Fig. 4e).

Similarly to middle-aged mice, ovariectomized young adult mice spent less time in running wheels (*p* = 0.0058), covered shorter distances (*p* = 0.0032), and had lower average speeds (*p* < 0.0001). Ovariectomy in young adult mice reduced the number of entries into the running wheel compared to sham-operated animals (*p* = 0.0052) (Fig. 4a-d). As middle-aged mice, all young adult animals were mainly active at night. Also, the main effect of ovariectomy on the reduction of running activity was observed. During the night hours, from 8:00 p.m. to 5:00 a.m. (8:00 p.m. − 2:00 a.m. (*p* < 0.0001), 3:00 a.m. (*p* = 0.0001), 4:00 a.m. (*p* = 0.0314), 5:00 a.m. (*p* = 0.0162)), animals in the SHAM/VWR_Y group ran nearly three times more kilometers than those in the OVX/VWR_Y group (Fig. 4f).

### Voluntary wheel running and metformin treatment modulate NLRP3 inflammasome expression in the frontal cortex of middle-aged female mice

**Fig. 5:**
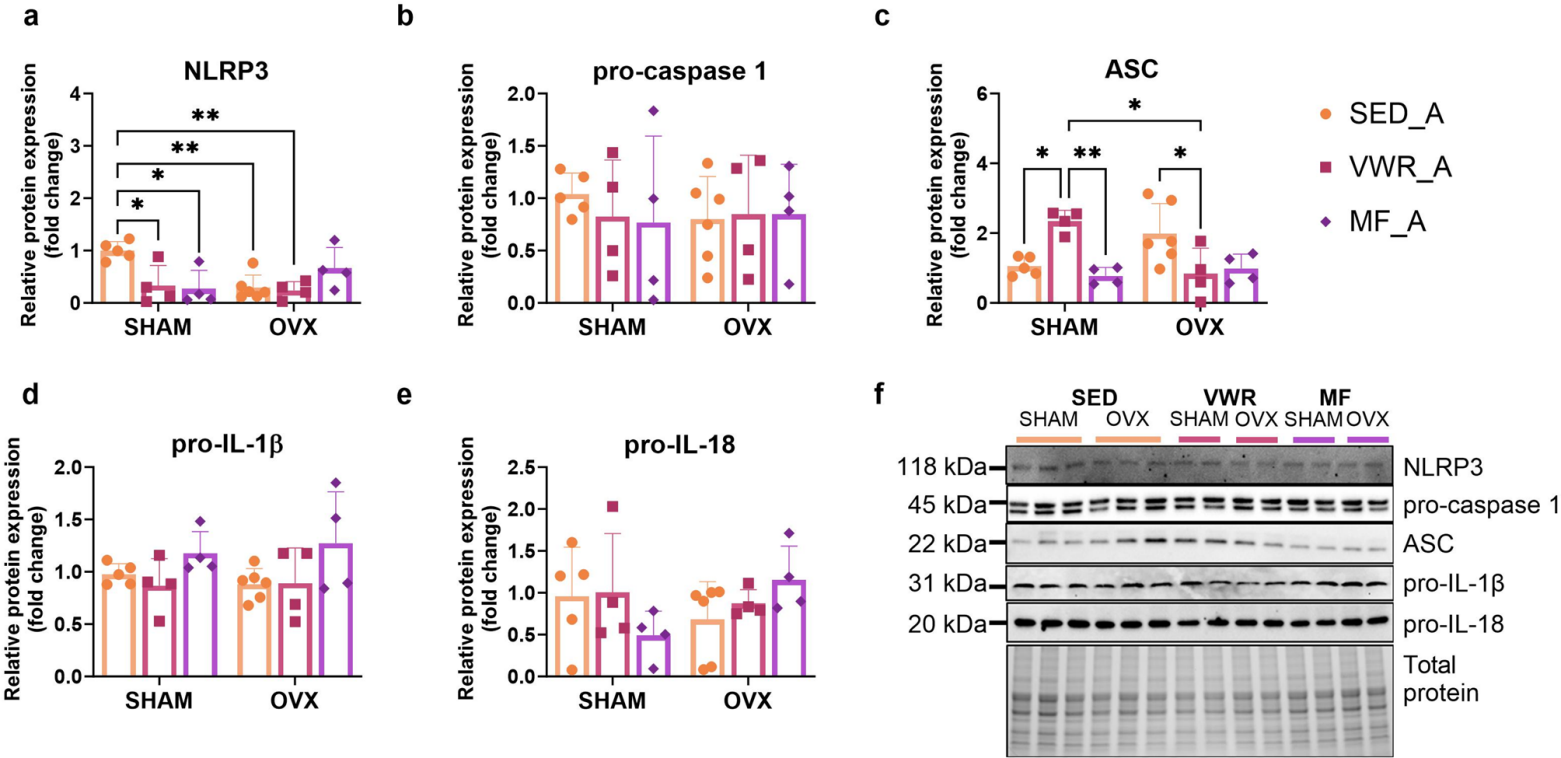
Effects of six weeks of voluntary wheel running and metformin treatment on NLRP3 inflammasome components in the frontal cortex of middle-aged (13-months-old, A) (**a**-**e**) mice, seven weeks after bilateral ovariectomy or sham operation. Representative images of Western blotting analysis (**f**). The samples were derived from the same experiment, and gels/blots were processed in parallel. Values are ​​presented as mean ± SD. Two-way ANOVA followed by Tukey’s multiple comparison test was performed. *p < 0.05, **p < 0.01. SHAM - sham operation, OVX - bilateral ovariectomy, SED - sedentary, VWR - voluntary wheel running, MF - metformin. n per group: SED_A (5–6), VWR_A (4), MF_A (4).

In middle-aged mice, voluntary wheel running and metformin treatment led to changes in the cortical expression level of NLRP3, ASC, and pro-IL-1β. Sham-operated mice, subjected to voluntary wheel running (SHAM/VWR_A) (*p* = 0.0270) and metformin (SHAM/MF_A) (*p* = 0.0137) had decreased NLRP3 protein expression compared to sedentary animals (SHAM/SED_A). Additionally, long-term ovariectomy (OVX/SED_A) (*p* = 0.0070) or ovariectomy combined with voluntary wheel running (OVX/VWR_A) (*p* = 0.0092) significantly reduced its expression compared to the SHAM/SED_A group. In animals after the sham operation, voluntary wheel running (SHAM/VWR_A) increased the expression level of ASC compared to the control sedentary (SHAM/SED_A) (*p* = 0.0260) and metformin-treated (SHAM/MF_A) (*p* = 0.0083) groups. Conversely, in ovariectomized middle-aged mice, voluntary wheel running (OVX/VWR_A) decreased ASC protein expression compared to sedentary animals (OVX/SED_A) (*p* = 0.0457) (Fig. 5a-c, f). There were no significant differences in pro-IL-1β expression levels between the experimental groups. No changes in pro-caspase 1 and pro-IL-18 expression levels were noted (Fig. 5d, e).

**Fig. 6:**
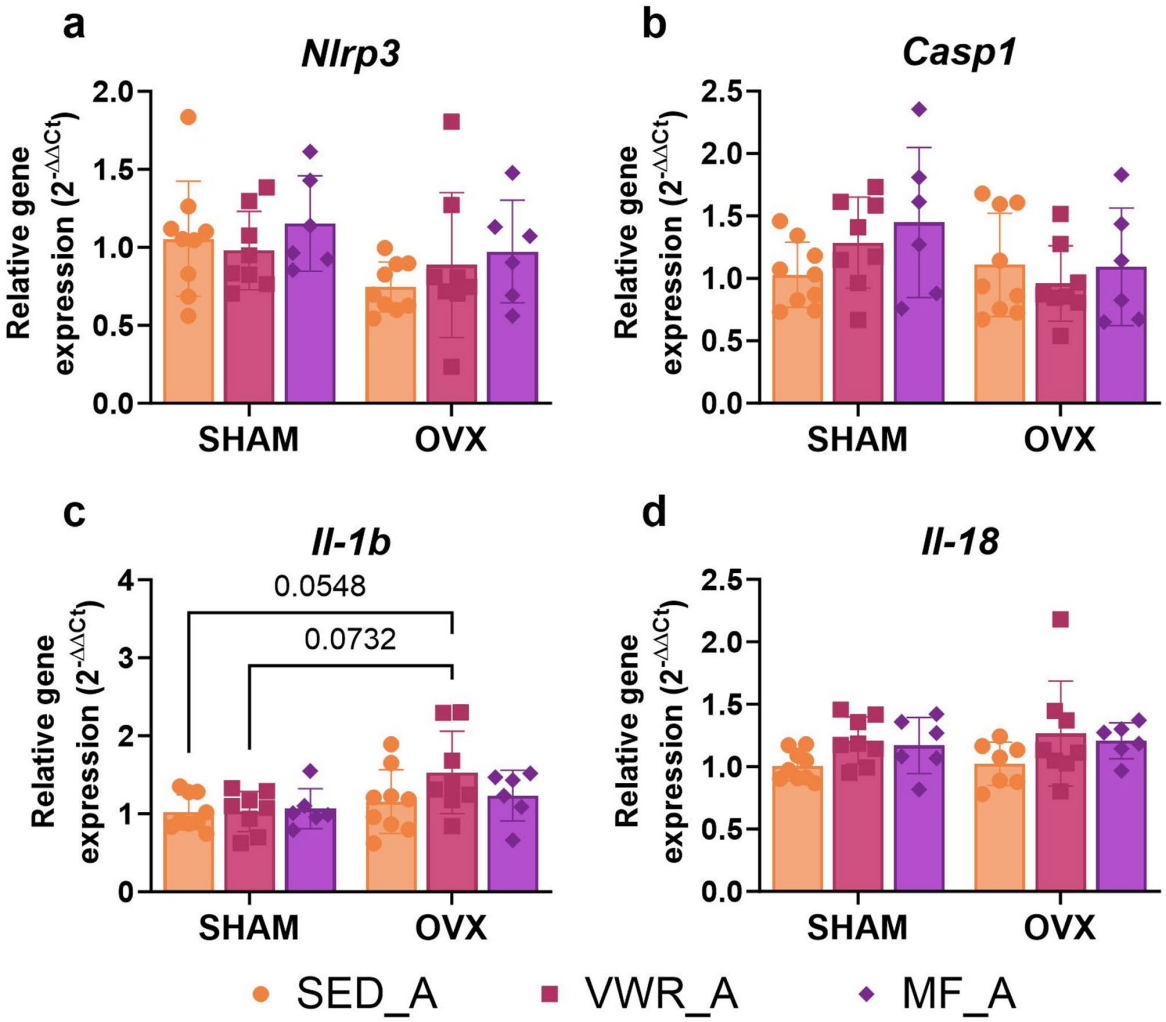
Effects of six weeks of voluntary wheel running and metformin treatment on the mRNA expression of NLRP3 inflammasome components in the frontal cortex of middle-aged (13-months-old, A) (**a**-**d**) mice, observed seven weeks after bilateral ovariectomy or sham operation. Values are ​​presented as mean ± SD. Two-way ANOVA followed by Tukey’s multiple comparison test was performed. SHAM - sham operation, OVX - bilateral ovariectomy, SED - sedentary, VWR - voluntary wheel running, MF - metformin. n per group: SED_A (9), VWR_A (8), MF_A (6).

Ovariectomy significantly affected the *Il-1b* gene expression level. Additionally, interventions changed the *Il-18* gene expression level. No significant differences between experimental groups at the mRNA level were revealed. There was a tendency toward reduction in the level of *Il-1b* gene expression in running mice after ovariectomy (OVX/VWR_A) compared to animals that had undergone the sham operation without access to running wheels (SHAM/SED_A) (*p* = 0.0548) (Fig. 6c, d). No changes were found in the mRNA expression level of *Nlrp3* and *Casp1* genes (Fig. 6a, b).

**Fig. 7:**
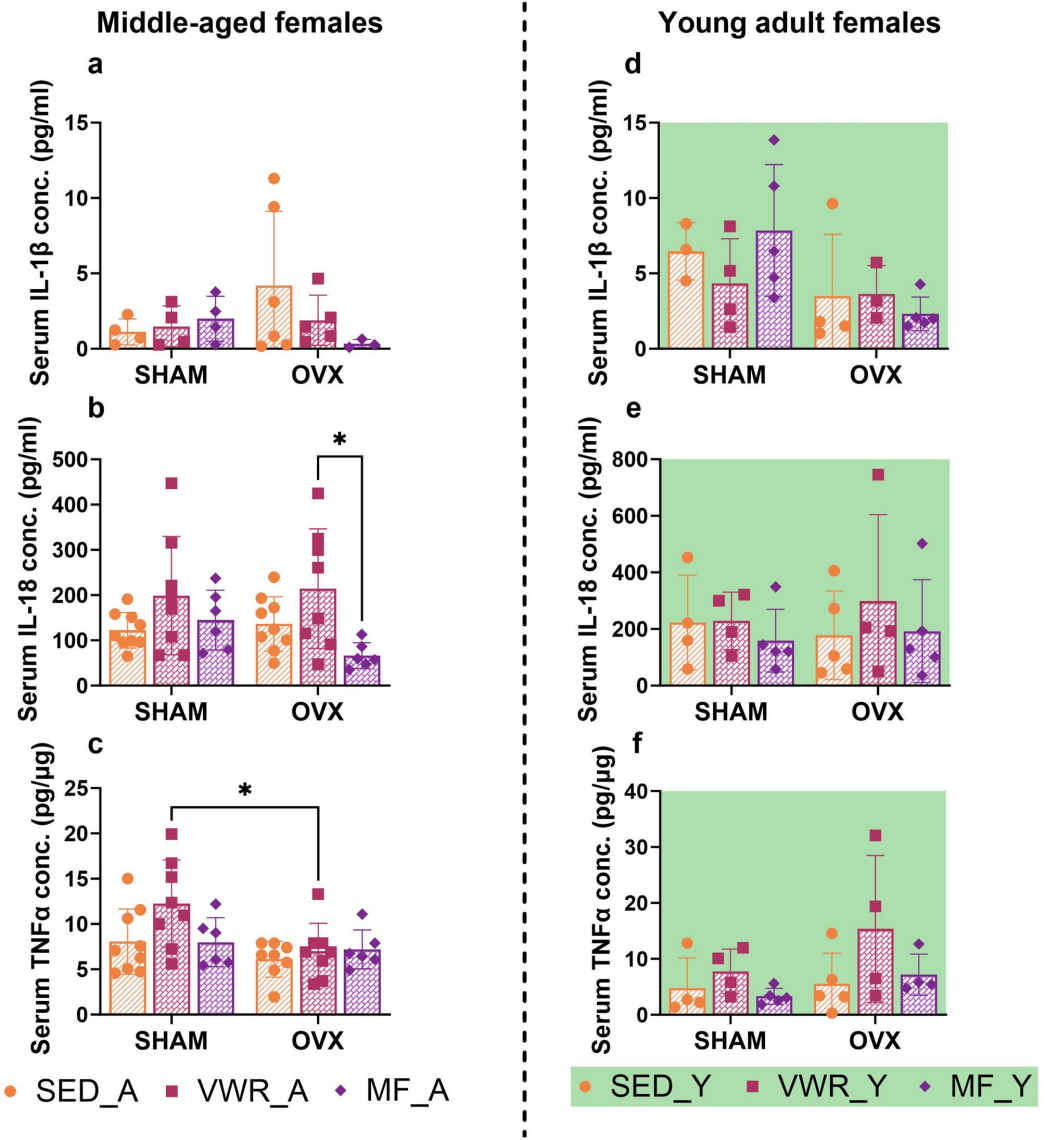
Effects of six weeks of voluntary wheel running and metformin treatment on serum pro-inflammatory cytokines concentration in middle-aged (13-month-old, A) (**a**-**c**) and young adult (2-month-old, Y) (**d**-**f**) mice, seven weeks after bilateral ovariectomy or sham operation. No changes in the concentration of IL-1β in the serum of middle-aged mice were observed (**a**). Among ovariectomized middle-aged animals, a decrease in serum IL-18 concentration after six weeks of metformin intake (OVX/MF_A) compared to voluntary running animals (OVX/VWR_A) was noted (**b**). Additionally, among the running animals, those that underwent sham operation had increased serum TNF-α concentrations compared to the ovariectomized group (**c**). No changes in serum IL-1β, IL-18, and TNF-α concentrations between young adult experimental groups were observed (**d**-**f**). Values are ​​presented as mean ± SD. Two-way ANOVA followed by Tukey’s multiple comparison test was performed. *p < 0.05. SHAM - sham operation, OVX - bilateral ovariectomy, SED - sedentary, VWR - voluntary wheel running, MF - metformin. Serum IL-1β conc. (n per group): SED_A (4), VWR_A (4), MF_A (4), SED_Y (3–4), VWR_Y (4), MF_Y (5). Serum IL-18, TNF-α conc. n per group: SED_A (9), VWR_A (8), MF_A (6), SED_Y (4–5), VWR_Y (4), MF_Y (5).

Metformin treatment lowered the serum IL-18 concentration in ovariectomized middle-aged mice (OVX/MF_A) as compared to voluntary running animals (OVX/VWR_A) (*p* = 0.0381) (Fig. 7b). Additionally, among the running animals, those that underwent sham operation had increased serum TNF-α concentrations compared to the ovariectomized group (SHAM/VWR_A vs. OVX/VWR_A: *p* = 0.0305) (Fig. 7c). No changes in the concentration of IL-1β in the serum of middle-aged mice were observed (Fig. 7a).

### Long-term ovariectomy and voluntary wheel running modulate the expression of proteins involved in NLRP3 inflammasome activation in young adult mice

**Fig. 8:**
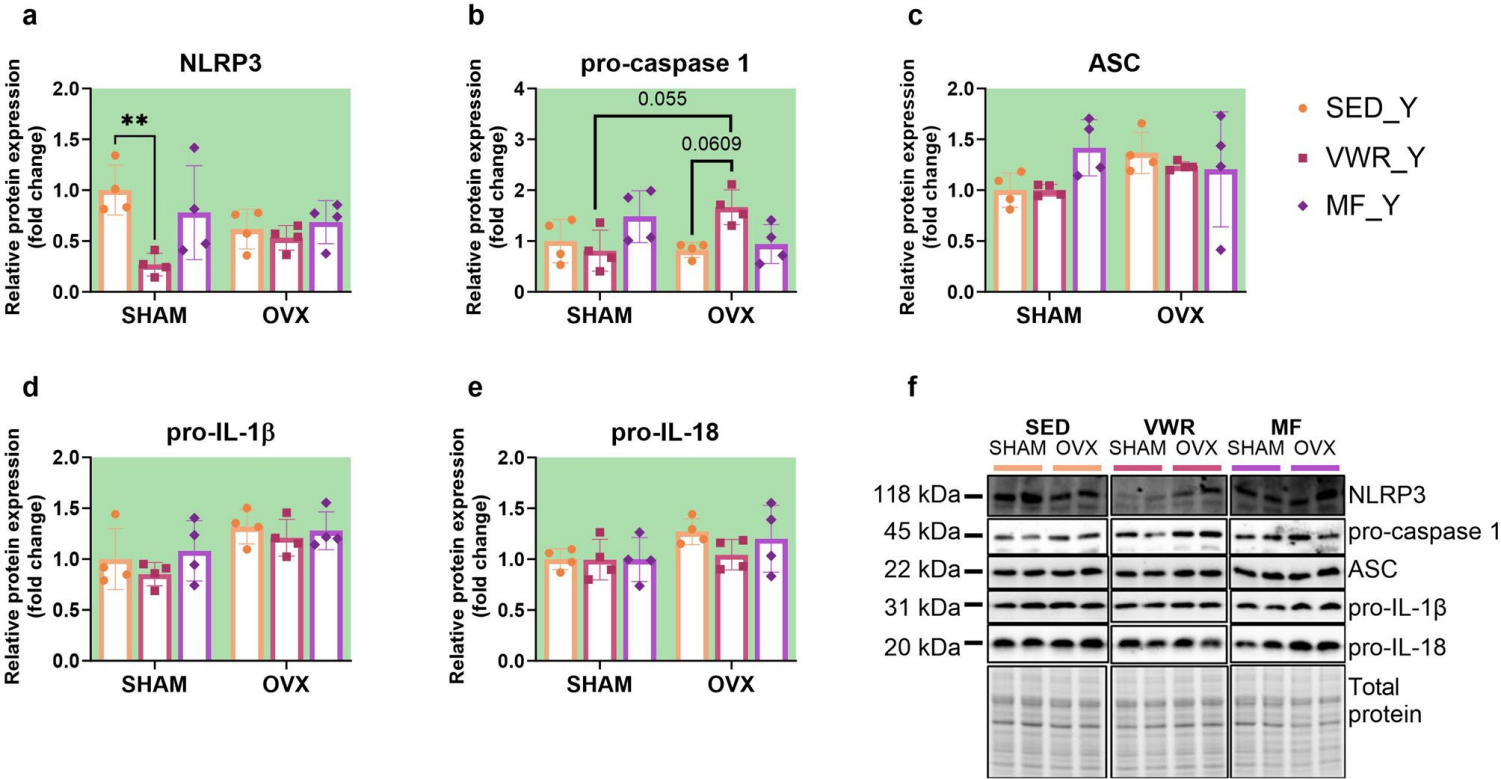
Effects of six weeks of voluntary wheel running and metformin treatment on NLRP3 inflammasome components in the frontal cortex of young adult (2-months-old, Y) (**a**-**e**) mice, seven weeks after bilateral ovariectomy or sham operation. Representative images of Western blotting analysis, cropped bands from different parts of the same gel and blots are shown (**f**). The samples were derived from the same experiment, and gels/blots were processed in parallel. Values are ​​presented as mean ± SD. Two-way ANOVA followed by Tukey’s multiple comparison test was performed. **p < 0.01. SHAM - sham operation, OVX - bilateral ovariectomy, SED - sedentary, VWR - voluntary wheel running, MF - metformin. n per group: SED_Y (4), VWR_Y (4), MF_Y (4).

**Fig. 9:**
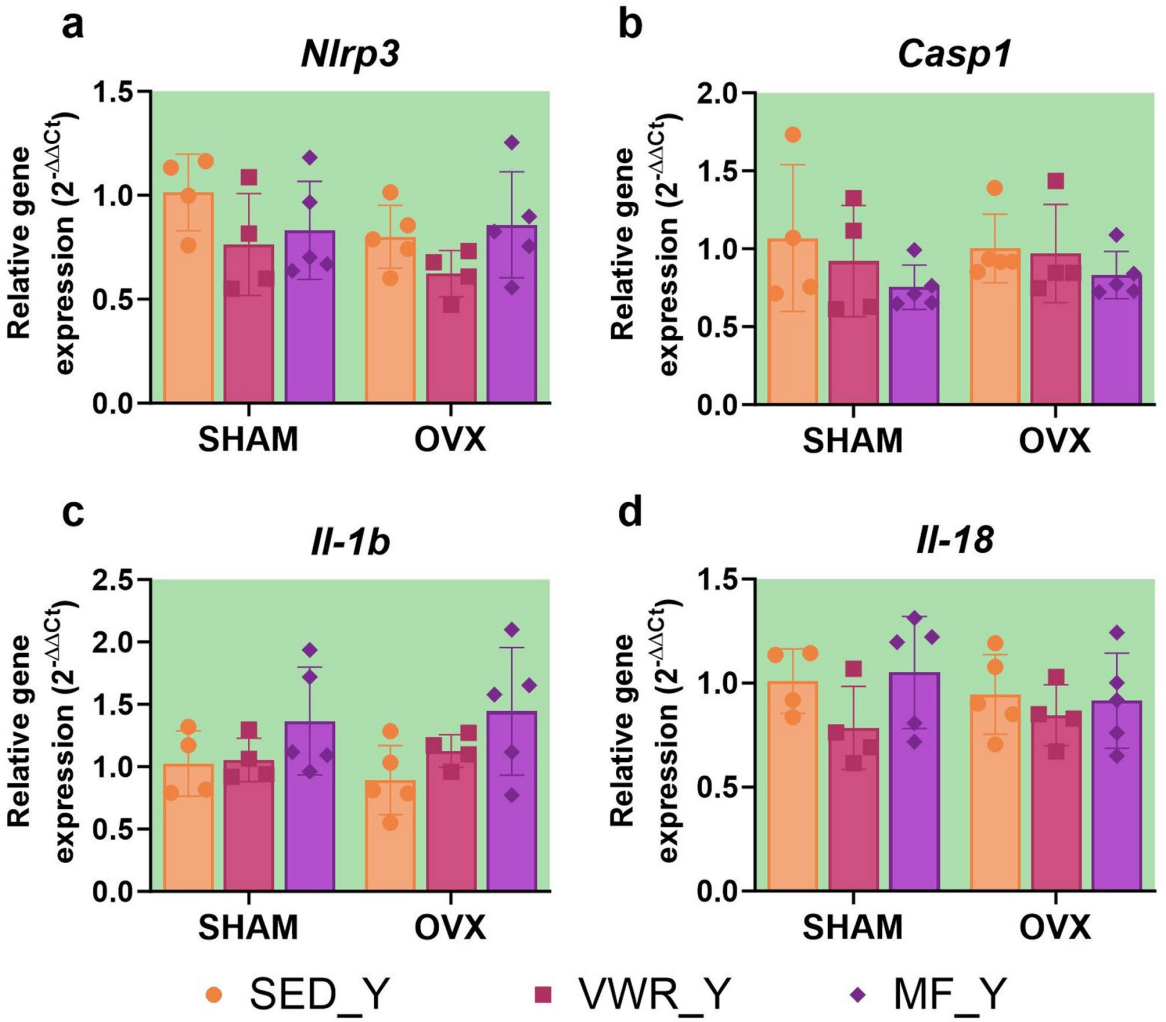
Effects of six weeks of voluntary wheel running and metformin treatment on the mRNA expression of NLRP3 inflammasome components in the frontal cortex of young adult (2-months-old, Y) (**a**-**d**) mice, observed seven weeks after bilateral ovariectomy or sham operation. Values are ​​presented as mean ± SD. SHAM - sham operation, OVX - bilateral ovariectomy, SED - sedentary, VWR - voluntary wheel running, MF - metformin. n per group: SED_Y (4–5), VWR_Y (4), MF_Y (5).

Six weeks of voluntary wheel running significantly decreased the expression level of NLRP3 protein in the frontal cortex of young adult sham-operated mice compared to sedentary control animals (SHAM/SED_Y vs. SHAM/VWR_Y: *p* = 0.0076) (Fig. 8a, f). Simultaneous exposure to long-term ovariectomy and voluntary running tended to upregulate pro-caspase 1 expression in comparison to running animals after sham operation (SHAM/VWR_Y vs. OVX/VWR_Y: *p* = 0.055). Tendency to increase pro-caspase 1 expression was also observed among ovariectomized animals (OVX/SED_Y vs. OVX/VWR_Y: *p* = 0.0609) (Fig. 8b). After seven weeks of the experiment, ovariectomy affected the expression level of pro-IL-1β and pro-IL-18. However, it did not induce differences between the experimental groups (Fig. 8d, e). No changes in ASC expression were noted (Fig. 8c). The main effect of voluntary wheel running or metformin treatment on mRNA expression of the *Il-1b* gene, with no changes between groups was revealed (Fig. 9c). The gene expression levels of *Nlrp3*, *Casp1*, and *Il-18* did not change (Fig. 9a, b,d). In contrast to middle-aged females, ovariectomy in young adult mice affected serum concentrations of IL-1β, showing no significant differences between groups (Fig. 7d). There were no changes in serum IL-18 and TNF-α concentrations (Fig. 7e, f).

### Long-term ovariectomy and voluntary wheel running affect the expression level of genes involved in NF-κB signaling pathway regulation in the frontal cortex of middle-aged mice

**Fig. 10:**
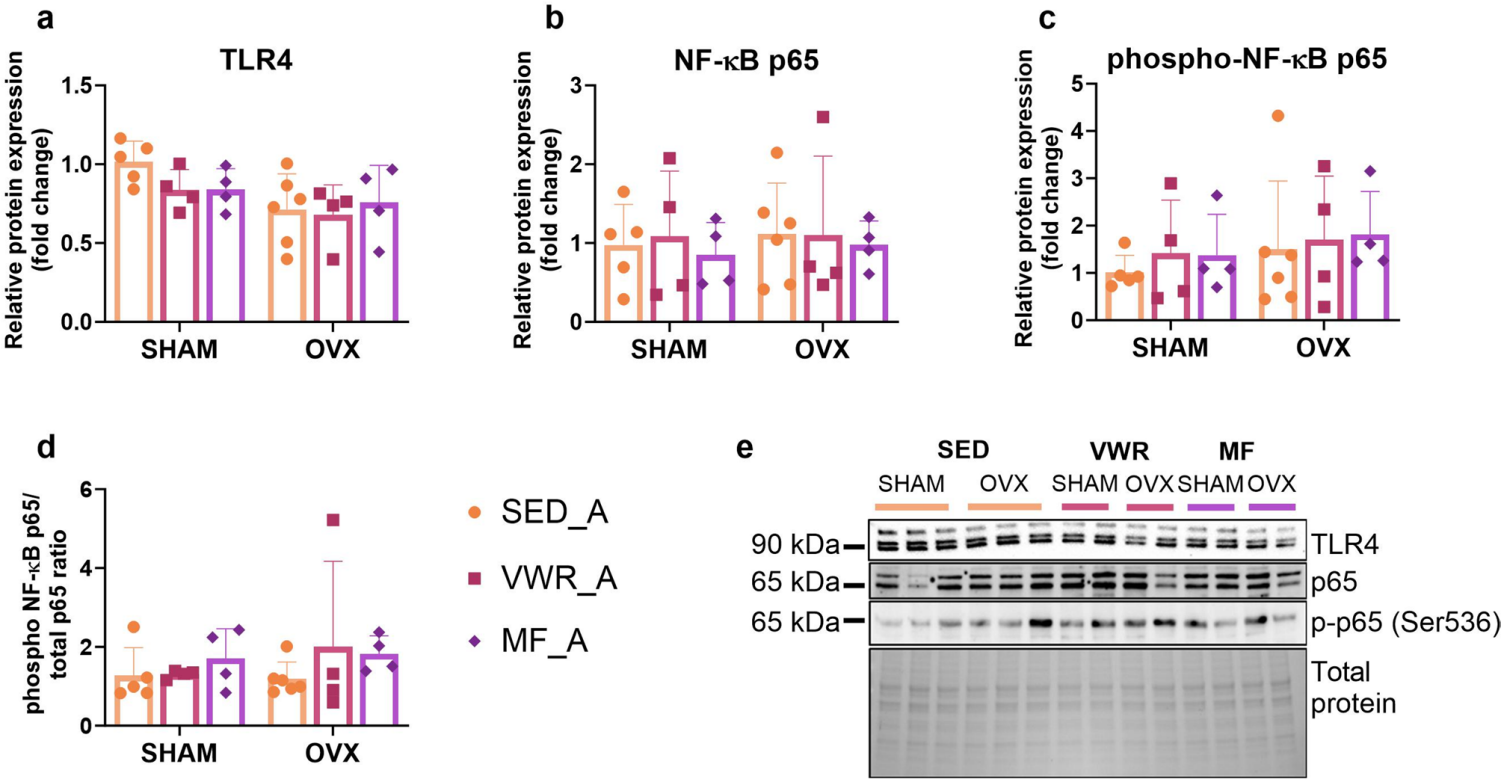
Effect of six weeks of voluntary wheel running and metformin treatment on TLR4/NF-κB signaling pathway in the frontal cortex of middle-aged (13-month-old, A) (**a**-**d**) mice, seven weeks after bilateral ovariectomy or sham operation. Representative images of Western blotting analysis (**e**). The samples were derived from the same experiment, and gels/blots were processed in parallel. Values are ​​presented as mean ± SD. SHAM - sham operation, OVX - bilateral ovariectomy, SED - sedentary, VWR - voluntary wheel running, MF - metformin. n per group: SED_A (5–6), VWR_A (4), MF_A (4).

**Fig. 11:**
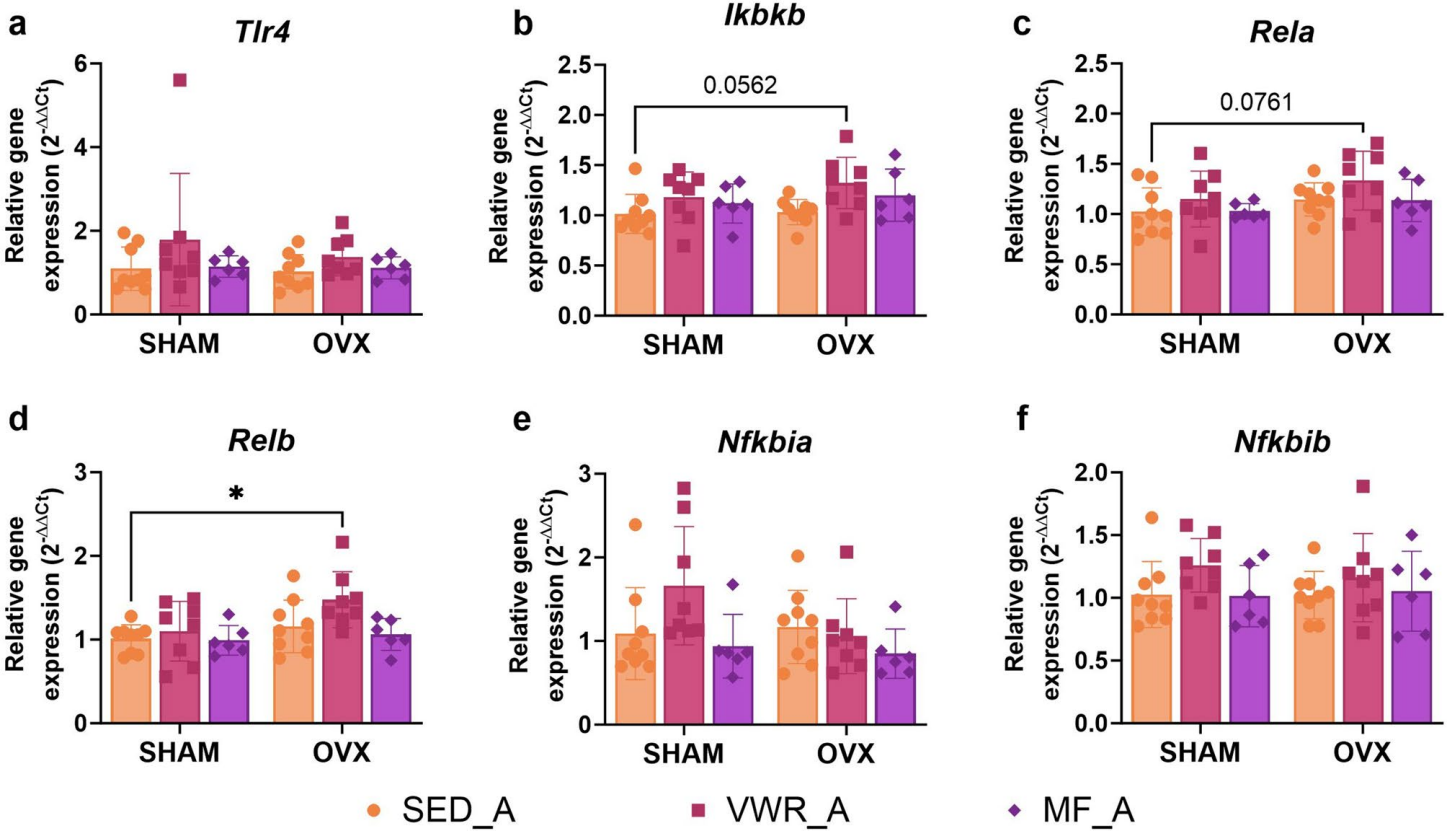
Effect of six weeks of voluntary wheel running and metformin treatment on TLR4/NF-κB signaling pathway mRNA expression in the frontal cortex of middle-aged (13-month-old, A) (**a**-**f**) mice, seven weeks after bilateral ovariectomy or sham operation. Values are ​​presented as mean ± SD. Two-way ANOVA followed by Tukey’s multiple comparison test was performed. *p < 0.05. SHAM - sham operation, OVX - bilateral ovariectomy, SED - sedentary, VWR - voluntary wheel running, MF - metformin. n per group: SED_A (9), VWR_A (8), MF_A (6).

Among the analyzed proteins involved in the NF-κB signaling pathway in the frontal cortex of middle-aged mice, the TLR4 expression was significantly changed by ovariectomy. However, there were no differences in the TLR4 expression levels between the experimental groups (Fig. 10a). Also, no changes were observed in the expression level of the total NF-κB p65, phospho-NF-κB p65 (Ser536) (p-p65), and in the p-p65/p65 ratio (Fig. 10b-e). Ovariectomy significantly affected the expression level of the *Rela* and *Relb* genes. Also, we observed that voluntary wheel running altered the *Relb* and *Ikbkb* genes expression (Fig. 11b-d). In middle-aged females after bilateral ovariectomy, voluntary wheel running (OVX/VWR_A) significantly increased *Relb* expression compared to the sedentary sham-operated animals (SHAM/SED_A) (*p* = 0.0147) (Fig. 11d). Similarly, voluntary wheel running and bilateral ovariectomy (OVX/VWR_A) tended to increase *Rela* expression level compared to the SHAM/SED_A control group (*p* = 0.0761), but also *Ikbkb* expression level compared to the SHAM/SED_A group (*p* = 0.0562) and OVX/SED_A (*p* = 0.0843), but without reaching significant level (Fig. 11b, c). No changes were noted in the *Tlr4*, *Nfkbia*, and *Nfkbib* mRNA expression levels (Fig. 11a, e,f).

### Voluntary wheel running and metformin change expression level of proteins of TLR4/NF-κB p65 signaling pathway in the frontal cortex of young adult female mice

**Fig. 12:**
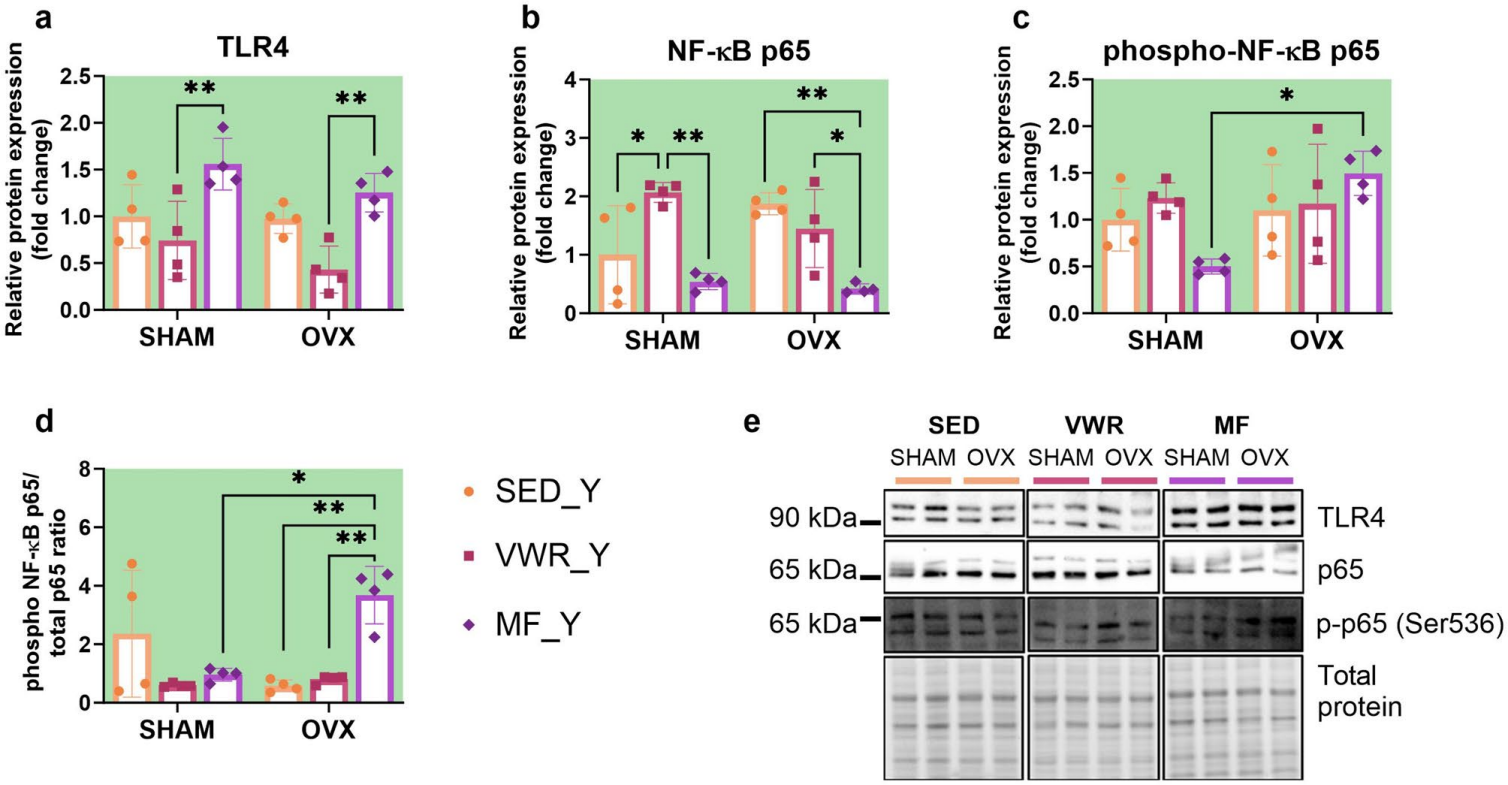
Effect of six weeks of voluntary wheel running and metformin treatment on TLR4/NF-κB signaling pathway in the frontal cortex of young adult (2-months-old, Y) (**a**-**d**) mice, seven weeks after bilateral ovariectomy or sham operation. Representative images of Western blotting analysis, cropped bands from different parts of the same gel and blots are shown (**e**). The samples were derived from the same experiment, and gels/blots were processed in parallel. Values are ​​presented as mean ± SD. Two-way ANOVA followed by Tukey’s multiple comparison test was performed. *p < 0.05, **p < 0.01. SHAM - sham operation, OVX - bilateral ovariectomy, SED - sedentary, VWR - voluntary wheel running, MF - metformin. n per group: SED_Y (4), VWR_Y (4), MF_Y (4).

**Fig. 13:**
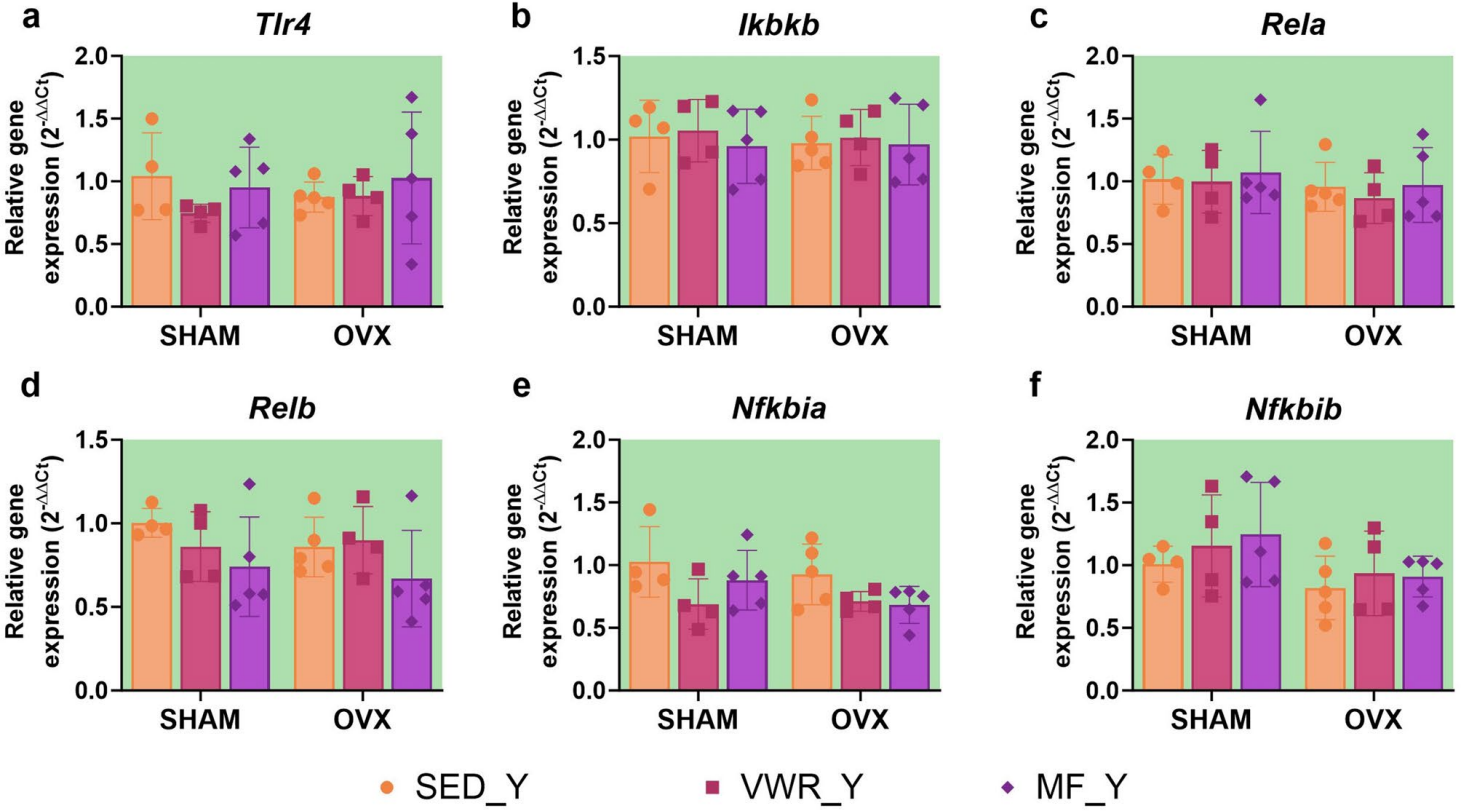
Effect of six weeks of voluntary wheel running and metformin treatment on TLR4/NF-κB signaling pathway mRNA expression in the frontal cortex of young adult (2-months-old, Y) (**a**-**f**) mice, seven weeks after bilateral ovariectomy or sham-operation. Values are ​​presented as mean ± SD. SHAM - sham operation, OVX - bilateral ovariectomy, SED - sedentary, VWR - voluntary wheel running, MF - metformin. n per group: SED_Y (4–5), VWR_Y (4), MF_Y (5).

Metformin changed the cortical expression of the TLR4 in young adult mice. In young adults, regardless of the operation type, metformin increased the expression level of TLR4 in comparison to animals subjected to voluntary wheel running (SHAM/VWR_Y vs. SHAM/MF_Y: *p* = 0.0091, OVX/VWR_Y vs. OVX/MF_Y: *p* = 0.0085) (Fig. 12a). Voluntary wheel running and metformin treatment changed NF-κB p65 expression. Running enhanced its expression level in sham-operated animals (SHAM/SED_Y vs. SHAM/VWR_Y: *p* = 0.0374). Oppositely, metformin treatment reduced NF-κB p65 expression level in young adult ovariectomized animals compared to sedentary mice (OVX/SED_Y vs. OVX/MF_Y: *p* = 0.0031). SHAM/MF_Y and OVX/MF_Y mice had significantly lower expression level NF-κB p65 than running animals after the same operation (SHAM/VWR_Y vs. SHAM/MF_Y: *p* = 0.0019, OVX/VWR_Y vs. OVX/MF_Y: *p* = 0.0485) (Fig. 12b). Long-term ovariectomy elevated the level of phospho-NF-κB p65 (SHAM/MF_Y vs. OVX/MF_Y: *p* = 0.0154), as well as increased the phospho-NF-κB p65/total p65 ratio (SHAM/MF_Y vs. OVX/MF_Y: *p* = 0.0111) among metformin-treated young adult mice. Also, an enhancement of the protein ratio in young adult ovariectomized mice after metformin treatment was observed compared to sedentary and running groups (OVX/SED_Y vs. OVX/MF_Y *p* = 0.0034, OVX/VWR_Y vs. OVX/MF_Y *p* = 0.0068) (Fig. 12c-e). Voluntary wheel running and metformin significantly impacted *Nfkbia* gene expression, while ovariectomy changed *Nfkbib* gene expression level. However, mRNA expression did not differ between experimental groups (Fig. 13e, f). No changes were observed in *Tlr4*, *Ikbkb*, *Rela*,* and Relb* mRNA expression (Fig. 13a-d).

## Discussion

Aging has been associated with an increased risk of developing chronic diseases, including CNS disorders^68^. It is estimated that due to observed demographic changes, the percentage of women living in postmenopause will probably increase^69^. In animal models of menopause, it has been shown that ovarian hormones deficiency may enhance neuroinflammation processes, which eventually contribute to the development of neurodegenerative disorders^70,71^. Here, we performed an experiment employing a well-known surgical menopause model - ovariectomy, that mimics ovarian hormones deficiency state^72^. Unexpectedly, we did not note serum E2 changes after ovariectomy (see Supplementary Table 5, Table 6, Additional file 1), whereas similar observations were reported previously^73,74^. All ovariectomized mice had lower uterine weights and were in the metestrus/diestrus cycle phase (see Supplementary Table 7, Table 8, Additional file 1), which are typical characteristics in rodent models of surgical menopause^75,76^. In the other study, increased expression of NLRP3, caspase 1, and P2X7R in the hippocampus of adult mice was observed following ovariectomy. Furthermore, it also resulted in the upregulation of IL-1β and IL-18^77^. In the hippocampus of adult rats, ovariectomy may lead to increased expression of proteins involved in the activation of the NLRP3 inflammasome. Moreover, it could contribute to the upregulation of TLRs and caspase 8 expression, leading to the activation of the NF-κB signaling pathway^78,79^. Here, in middle-aged mice, we observed increased pro-caspase 1 and pro-IL-1β cortical expression and serum IL-1β concentration two weeks after ovariectomy. Similarly to our observations in the frontal cortex, the expression pattern of ASC, pro-caspase 1, and pro-IL-1β was previously reported in the hippocampus one month after ovariectomy^77,78^. In the frontal cortex of middle-aged mice, we showed that short-term ovariectomy increases the expression of the TLR4. An increase in the hippocampal expression of the TLR4 was previously noted in adult rodents up to ten weeks after ovariectomy, leading to the activation of NF-κB p65^77,80^. We did not observe changes in the cortical expression of proteins or genes involved in the activation of the NF-κB signaling pathway two weeks after ovariectomy. These results differ from previous reports, which may be due to researchers focusing solely on neuroinflammatory processes in the hippocampus and adult rodents. The differential responses to ovariectomy between brain structures have been previously reported. In the frontal cortex, but not in the hippocampus of 15-month-old female mice, reduced TNF-α, CXCL1, IL-10, IL-12p70, IFN-γ, and IL-6 have been shown after ovariectomy^81^.

A healthy lifestyle, including regular PA, was found to play a substantial role in improving the quality of life and maintaining health in postmenopause^82^. Similar to previous reports, ovariectomy has not altered the nocturnal activity, as evidenced by the animals remaining primarily active at night^83^. Ovariectomized middle-aged and young adult mice covered a shorter distance on running wheels than animals that underwent a sham operation in the dark phase. Such differences were not observed in the light phase. Previously, similar observations were noted when ovariectomy reduced spontaneous PA in the dark phase, while it had no effect in the light phase^84^. Typically, mice with free access to running wheels cover a total distance of 4 to 20 km per day^85^. It was previously reported that ovariectomy^86,87^ or ovarian senescence^88^ reduces the level of PA. In the present study, during six weeks of voluntary running in middle-aged and young adult mice, ovariectomy decreased the covered distance by half. This change was associated with shorter time spent on running wheels and a reduced running speed. It has been previously shown that ovarian removal may indirectly promote apoptosis processes and subsequently contribute to the reduction of skeletal muscle mass^89^. Therefore, we could speculate that the observed ovariectomy-induced inactivity may be linked with increased apoptosis and a reduction in skeletal muscle mass.

The beneficial effect of PA on the CNS has been confirmed in both humans and rodents^22,24,28,30,37,39^. Previous reports indicated that high-intensity interval training and moderate-intensity continuous training inhibit the expression of NLRP3 inflammasome components and ischemic stroke-induced pyroptosis^33,90^. PA was also found to suppress the LPS-induced increased expression of P2X7R, NLRP3 inflammasome components, and proinflammatory cytokines (IL-6, IL-1β, and TNF-α) in the hippocampus and the cerebral cortex^38^. In a model of AD, the expression of proinflammatory cytokines and the activation of the NLRP3 inflammasome and glial cells in the prefrontal cortex and the hippocampus were reduced as a result of PA intervention^30,31^. According to our knowledge, the modulating effect of PA on neuroinflammation processes in the menopause model was demonstrated twice^91,92^. Ovariectomy-induced reduction in ERβ expression in the hippocampus of adult mice was associated with an increased expression of NLRP3, caspase 1, IL-1β, and IL-18^92^. Moreover, the expression of TNF-α, IL-1β, and COX-2 was enhanced while the BDNF expression was lowered in the cortex after subjecting adult rats to ovariectomy^91^. Conversely, introducing PA for four weeks was determined to mitigate the changes induced by ovariectomy in both the hippocampus and the cortex^91,92^. Similarly, six weeks of voluntary wheel running of middle-aged and young adult sham-operated mice downregulated the cortical expression of NLRP3 compared to sedentary control animals. Also, reduced NLRP3 expression was noted in ovariectomized, running middle-aged, but not in young adult, mice. Surprisingly, unlike previous studies, we did not find NLRP3 upregulation after long-term ovariectomy in middle-aged and young adult mice. In contrast, ovariectomy has been show to lower cortical expression of NLRP3 in middle-aged mice. In middle-aged animals, we observed changes in ASC expression. In the sham-operated mice, voluntary running increased ASC expression, whereas a decrease in the expression of this protein was noted in ovariectomized animals. Unlike young adults, among running middle-aged mice, those sham-operated had higher serum TNF-α concentrations than ovariectomized ones. We noted an increased level of *Relb* expression in the frontal cortex of ovariectomized middle-aged mice subjected to voluntary wheel-running compared to sham-operated sedentary animals. In primary astrocytes, RelB expression reduces IL-1β-induced mRNA expression of proinflammatory cytokines IL-1β, IL-6, and IL-8^93^. Also, in astrocyte-specific RelB-deficient mice, an increase in IL-1β and IL-6 expression was demonstrated after LPS stimulation^93^. It has been shown that E2 inhibits the expression of genes associated with inflammation regulation by blocking the translocation of RelB to the cell nucleus of the macrophages, thereby inducing a proinflammatory response^94^. Therefore, it may be assumed that the increase in RelB expression inhibits neuroinflammation processes, reducing the expression of proinflammatory cytokines in vitro and in vivo. It may be suggested that voluntary running induces anti-inflammatory effects in the frontal cortex of middle-aged mice as a result of changes in the *Relb* mRNA expression level. However, after PA, no significant differences between groups in the expression of studied genes (*Tlr4*, *Ikbkb*, *Rela*, *Nfkbia*, *Nfkbib*), and proteins involved in the NF-κB signaling pathway were observed, further supporting this thesis. Moreover, *Relb* upregulation was observed in ovariectomized middle-aged mice with running activity levels lower than sham-operated animals, which may suggest the role of training intensity in the context of modulating neuroinflammation. We have not checked whether the intensity of PA could determine the regulation of neuroinflammation processes, which might be considered a limitation of the present study.

Introducing regular PA is not always possible for patients, mainly due to health restrictions. Therefore, the application of EMs has been proposed, the action of which is to induce beneficial changes similar to those observed as a result of PA. PA was established to lead to changes at the molecular, cellular, and behavioral levels, while using an EM it is not possible to trigger the same broad-spectrum effects. Nonetheless, it is suggested that it might exert a beneficial effect on regulating selected processes, including neuroinflammation. Here, metformin has been proposed as an EM, which potentially affects the regulation of neuroinflammation processes in pathological conditions^95–98^. While comparing the effects of the two interventions, middle-aged mice that underwent a sham operation showed decreased NLRP3 expression following running and metformin treatment compared to control animals. Additionally, while metformin treatment reduced serum IL-18 concentration in middle-aged ovariectomized mice, no such effect was observed with voluntary wheel running. So far, we have not found studies reporting metformin effects on neuroinflammation processes after ovariectomy in rodents. However, it was shown in previous reports that metformin reduces inflammation and antioxidant activity, as well as reduces depressive and anxiety-related behaviors^99,100^. The ability to regulate neuroinflammation and apoptosis processes by metformin was observed in the hippocampus and the cerebral cortex of AD rodent models and aged rodents^101–103^. Also, after stroke, it reduced the expression of proinflammatory cytokines (IL-1β, IL-4, IL-6, TNF-α) in the rat striatum^57^. It is suggested that the anti-inflammatory effect of metformin results from the activation of AMPK, which consequently may inhibit the activation of the NF-κB signaling pathway, NLRP3 inflammasome, and increase BBB integrity^55,56^.

While no differences between middle-aged groups in NF-κB p65 expression were shown, we noted changes in young adult animals. In young adult mice, regardless of the operation type, metformin upregulated TLR4 expression levels concerning running animals. Metformin further decreased NF-κB p65 levels among ovariectomized mice compared to sedentary and active young adults. Conversely, voluntary wheel running in sham-operated mice increased its expression compared to control sedentary and metformin-treated young adults. Moreover, in metformin-treated young adults, those ovariectomized had higher p-p65 levels than sham-operated mice. Also, the p-p65/p65 ratio was increased in ovariectomized mice subjected to metformin, suggesting that metformin may promote cortical NF-κB p65 signaling pathway activation in young adult ovariectomized mice. In contrast, metformin has been previously reported to reduce the activation of the NF-κB signaling pathway and expression of proinflammatory cytokines in PD mice^104^. As ERs are well-known regulators of NF-κB activation^105^, we can speculate that the observed effects of metformin in young adults, but not in middle-aged mice, might result from the age-related decrease in ER activity^58^ and its dysregulation after ovariectomy^106^. Notably, there is growing evidence suggesting that the actions of metformin may depend on the dose and the actual reached concentrations. As metabolic changes occur with aging, the frontal cortex or serum metformin level measurement would provide additional insight into the differential response to the same dose of metformin in both studied aged groups. However, we have not performed a metformin evaluation, which may be considered a limitation of the study^107^.

## Conclusions

Our results may indicate a modulatory effect of voluntary wheel running and metformin treatment on neuroinflammation in the frontal cortex with varying effects that seem age-related and/or ovarian status-dependent. Even though ovariectomy reduced running activity in both studied age groups, a decrease in NLRP3 expression was observed in middle-aged mice following PA, while no similar effects were seen in young adult mice. Although we noted a decrease in NLRP3 expression levels, we cannot conclusively state that PA inhibits NLRP3 inflammasome activation. However, it may be hypothesized that responsiveness to PA and metformin treatment in young adult mice could be mainly associated with the NF-κB signaling pathway. Furthermore, the obtained results indicate that ovariectomy might increase the susceptibility of young adult mice to metformin. Our results further support the assumptions that neuroinflammation during the postmenopausal period is not only disrupted by ovarian status but also influenced by aging.

## Electronic supplementary material

Below is the link to the electronic supplementary material.

Supplementary Material 1

Supplementary Material 2

Supplementary Material 3

## Data Availability

The datasets used and/or analyzed during the current study are available from the corresponding author on reasonable request.

## Abbreviations

AD: Alzheimer’s Disease
AMPK: AMP-activated protein kinase
ASC: Apoptosis-associated speck-like protein containing a CARD
BBB: Blood-brain barrier
CNS: Central nervous system
E2: 17-β-estradiol
EM: Exercise mimetic
ER: Estrogen receptor
IL: Interleukin
MF: Metformin
NFκB: Nuclear factor kappa-light-chain-enhancer of activated B cells
NLRP3: Nucleotide-binding oligomerization domain-like receptor (NLR) family pyrin domain containing 3
OVX: Bilateral ovariectomy
PA: Physical activity
PD: Parkinson’s Disease
SHAM: Sham operation
TLR: Toll-like receptor
TNF: Tumor necrosis factor
VWR: Voluntary wheel running
