# Supplementary material for: Age-dependent neuroinflammation response to voluntary wheel running and Metformin treatment in the frontal cortex of ovariectomized female mice
Source: Sci Rep. 2025 Jul 21;15:26382. doi: 10.1038/s41598-025-10014-0 (PMC12280059; doi:10.1038/s41598-025-10014-0)
Supplement: Supplementary file 1 — Supplementary Material 1 [file 41598_2025_10014_MOESM1_ESM.pdf]

Additional File 1

Age-dependent neuroinflammation response to voluntary wheel running and metformin treatment in the frontal cortex of ovariectomized female mice

Konstancja Grabowska[1,2*], Mateusz Grabowski[2], Julia Morys[2], Edyta Olakowska[1], Andrzej Małecki[2], Jarosław J Barski[1], and Marta Nowacka-Chmielewska[2]

[1]Department of Physiology, Faculty of Medical Sciences in Katowice, Medical University of Silesia, Poland

[2]Laboratory of Molecular Biology, Institute of Physiotherapy and Health Sciences, Academy of Physical Education, Katowice, Poland

* Corresponding author: konstancja.grabowska@sum.edu.pl

keywords: neuroinflammation, NLRP3 inflammasome, voluntary wheel running, metformin, ovariectomy, physical activity

Supplementary Table 1, Additional file 1. Characteristics of the standard chow (Labofeed B, Wytwórnia Pasz Morawki) used in the experiment.

**Labofeed B composition (per kg)**			
Protein (g)	175	Total calories (kcal)	2747
Soybean meal (%)	8	Protein (kcal, %)	25
Potato protein (%)	3	Fat (kcal, %)	8
Feed yeast (%)	3	Carbohydrates (kcal, %)	67
Fat (g)	2.8		
Fiber (g)	70		
Starch (g)	330		
Ash (g)	57		
Calcium (g)	9.5		
Phosphorus (g)	6.5		
Magnesium (g)	3		
Potassium (g)	7.5		
Sodium (g)	1.9		
Sulfur (g)	1.9		
Iron (mg)	144		
Manganese (mg)	50		
Zink (mg)	50		
Copper (mg)	11		
Iodine (mg)	0.2		
Selenium (mg)	0.4		
Vitamin A (UI)	12000		
Vitamin D3 (UI)	800		
Vitamin E (mg)	78		
Vitamin K3 (mg)	2.4		
Vitamin B1 (mg)	8		
Vitamin B2 (mg)	7		
Vitamin B6 (mg)	11		
Vitamin B12 (mg)	42		
Pantothenic acid (mg)	25		
Folic acid (mg)	2		
Biotin (mg)	0.3		

Nicotinic acid (mg)	94
Choline (mg)	1900
Lysine (g)	9
Methionine+cysteine (g)	6.3
Tryptophan (g)	2
Threonine (g)	6
Isoleucine (g)	6
Leucine (g)	12
Valine (g)	8
Histidine (g)	4
Arginine (g)	10
Phenylalanine (g)	7
Tyrosine (g)	5.5
Betaine (g)	17

Supplementary Table 2, Additional file 1. Characteristics of antibodies used in the experiment.

Antibody	Producer (cat. no.)	Final concentration
TLR4 Polyclonal Antibody	Invitrogen, PA5-23124	2 µg/mL
Anti-NF-kB p65 antibody	Abcam, ab16502	1 µg/mL
Anti-NF-kB p65 (phospho S536) antibody	Abcam, ab86299	0.2 µg/mL
Anti-NLRP3 antibody [EPR23073-96]	Abcam, ab270449	0.5 µg/mL
ASC/TMS1 (D2W8U) Rabbit mAb	Cell Signaling, 67824	750-fold dilution
Anti-pro Caspase-1 + p102 + p12 antibody [EPR16883]	Abcam, ab179515	2 µg/mL
Anti-IL-1 beta antibody [EPR23851-127]	Abcam, ab254360	1 µg/mL
IL-18 (E8P5O) Rabbit mAb	Cell Signaling, 57058S	1000-fold dilution

Supplementary Table 3, Additional file 1. List of primers used in the experiment.

Gene	Sequence
Hprt1	F: CAGTCCCAGCGTCGTGATTAG<br>R: GTGATGGCCTCCCATCTCCTT
Gapdh	F:CAACTCCCTCAAGATTGTCAGCAA<br>R:GGCATGGACTGTGGTCATGA
Tlr4	F: TGCCACCAGTTACAGATCGTC<br>R: TTTGCTGAGTTTCTGATCCATGC
Rela	F: GCCTCTGGCGAATGGCTTTA<br>R: TGCTTCGGCTGTTCGATGAT
Relb	F: CTTGGGTTCCAGTGACCTCTC<br>R: TGGTCCTGGAGACCGTTAGT
Ikbkb	F: CCCAAAGAACAGAGACCGCT<br>R: TAAGAGCCGATGCGATGTCA
Nfkbia	F: TGCAGGCCACCAACTACAAT<br>R: AAGAGCGAAACCAGGTCAGG
Nfkbib	F: GAGCCGAAGTGTGATGCGA<br>R: GATGTGCCTGAGAACCAAGC
Nlrp3	F: GCAGAGCCTACAGTTGGGTG<br>R: ACGCCTACCAGGAAATCTCG
Casp1	F: GGCACATTTCCAGGACTGACTG<br>R: GCAAGACGTGTACGAGTGGTTG
Il-1b	F: GTTCATCTCGGAGCCTGTAGTG<br>R: TGGACCTTCCAGGATGAGGACA
Il-18	F: GACAGCCTGTGTTCGAGGATATG<br>R: TGTTCTTACAGGAGAGGGTAGAC

Supplementary Table 4, Additional file 1. Relative gene expression level (fold change).

Gene	SHAM	OVX	p-value
IL-18	1.01 ± 0.14	1.08 ± 0.42	0.7236[a]
Nlrp3	1.04 ± 0.29	0.94 ± 0.22	0.5727[a]
Il-1b	1.04 ± 0.35	0.96 ± 0.29	0.6814[a]
Casp1	1.01 ± 0.18	1.03 ± 0.61	0.9646[a]
Tlr4	1.04 ± 0.32	1.23 ± 0.35	0.3907[a]
RelA	1.02 ± 0.19	0.99 ± 0.23	0.8919[a]
RelB	1.07 ± 0.45	1.27 ± 1.14	0.6905[b]
Nfkbia	1.02 ± 0.21	1.21 ± 0.51	0.4628[a]
Nfkbib	1.03 ± 0.27	0.78 ± 0.25	0.1743[a]
IKKIB	1.04 ± 0.33	0.85 ± 0.31	0.6905[b]

Relative fold expression. Values are presented as the mean (± SD) of n = 5 per group (SHAM, OVX). [a]Unpaired t-test with Welch's correction, [b]Mann-Whitney test.

Supplementary Table 5, Additional file 1. Average serum 17-β-estradiol concentration and average uterine mass of middle-aged animals two weeks after operations.

	SHAM	OVX
**17-β-estradiol – Day 0 (pg/ml)**	32.65 ± 14.8	28.55 ± 11.32
**17-β-estradiol – Day 14 (pg/ml)**	31.69 ± 6.83	30.15 ± 7.47
**Uterus mass (mg/g body mass)**	3.52 ± 1.08	1.58 ± 0.32*

Values are presented as mean ± SD. Unpaired t-test with Welch's correction: SHAM vs. OVX, *p < 0.05 (Uterus mass). SHAM - sham operation, OVX - bilateral ovariectomy. n = 5 per group.

Supplementary Table 6, Additional file 1. Average serum 17-β-estradiol (E2) concentrations and average uterine mass of middle-aged and young adult animals seven weeks after operations.

	SHAM_A	OVX_A	SHAM_Y	OVX_Y
**E2 – Day 0 (pg/ml)**	29.64 ± 7.56	29.29 ± 10.3	48.6 ± 36.75	55.51 ± 43.97
**E2 – Day 49 (pg/ml)**	33.72 ± 8.94	29.28 ± 11.31	49.79 ± 37.69	55.93 ± 41.81
**Uterus mass (mg/g b.m.)**	5.15 ± 1.82	0.84 ± 0.21****	3.01 ± 1.16	1.13 ± 0.99^^^

Unpaired t-test with Welch's correction: SHAM (sham operation)_A vs. OVX (bilateral ovariectomy)_A, ****p<0.0001, Mann-Whitney test: SHAM_Y vs. OVX_Y, ^^^p<0.001. Mean ± SD. A (middle-aged): n = 23/group, Y (young adult): n = 13-14/group.

Supplementary Table 7, Additional file 1. The percentage distribution of the estrus cycle phases of middle-aged animals on the operation day and two weeks after operations.

	**Day 0**	**Day 14**	
	**ALL ANIMALS**	**SHAM**	**OVX**
**PROESTRUS**	30%	-	-
**ESTRUS**	20%	40%	-
**METESTRUS/DIESTRUS**	50%	60%	100%

SHAM - sham operation, OVX - bilateral ovariectomy. n = 10/all animals.

Supplementary Table 8, Additional file 1. The percentage distribution of the estrus cycle phases of middle-aged and young adult animals on the operation day and seven weeks after operations.

	**Day 0**		**Day 49**			
	**ALL MIDDLE-AGED ANIMALS**	**ALL YOUNG ADULT ANIMALS**	**SHAM_A**	**OVX_A**	**SHAM_Y**	**OVX_Y**
**PROESTRUS**	15.2%	22.2%	13.6%	-	5%	-
**ESTRUS**	32.6%	29.6%	27%	-	22%	-
**METESTRUS/ DIESTRUS**	52.2%	44.4%	59.4%	100%	73%	100%

SHAM - sham operation, OVX - bilateral ovariectomy. A (middle-aged): n = 46/all animals, Y (young adult): n = 27/all animals.
