# Supplementary material for: Age-dependent neuroinflammation response to voluntary wheel running and Metformin treatment in the frontal cortex of ovariectomized female mice
Source: Sci Rep. 2025 Jul 21;15:26382. doi: 10.1038/s41598-025-10014-0 (PMC12280059; doi:10.1038/s41598-025-10014-0)
Supplement: Supplementary file 2 — Supplementary Material 2 [file 41598_2025_10014_MOESM2_ESM.pdf]

Additional File 3

Age-dependent neuroinflammation response to voluntary wheel running and metformin treatment in the frontal cortex of ovariectomized female mice

Konstancja Grabowska[1,2*], Mateusz Grabowski[2], Julia Morys[2], Edyta Olakowska[1], Andrzej Małecki[2], Jarosław J Barski[1], and Marta Nowacka-Chmielewska[2]

[1]Department of Physiology, Faculty of Medical Sciences in Katowice, Medical University of Silesia, Poland

[2]Laboratory of Molecular Biology, Institute of Physiotherapy and Health Sciences, Academy of Physical Education, Katowice, Poland

* Corresponding author: konstancja.grabowska@sum.edu.pl

keywords: neuroinflammation, NLRP3 inflammasome, voluntary wheel running, metformin, ovariectomy, physical activity

Supplementary Table 1, Additional file 3. Statistical analysis results of the average serum 17-β-estradiol concentration of middle-aged females two weeks post-operations and middle-aged and young adult females seven weeks post-operations.

17-β-estradiol (pg/ml)	p-value
**Middle-aged female (2 weeks)**	0.1439[a]
**Middle-aged female (7 weeks)**	0.1232[b]
**Young adult females (7 weeks)**	0.9897[b]

Middle-aged (2 weeks): n = 5 per group, Middle-aged (7 weeks): n = 6 - 9 per group, Young adult (7 weeks): n = 4-5 per group. One-way ANOVA (a) or Kruskal-Wallis test (b) were performed

Supplementary Table 2, Additional file 3. The average daily activity characteristics from six weeks of voluntary wheel running.

	Middle-aged female	Young adult females
	SHAM vs. OVX p-value	SHAM vs. OVX p-value
**Time in the running wheel (h)**	0.0463[a]	0.0058[a]
**Total distance (km)**	< 0.0001[b]	0.0032[b]
**Speed (km/h)**	0.0477[b]	< 0.0001[b]
**Entries into the running wheel (no.)**	0.2343[b]	0.0052[a]

Values are presented as mean ± SD. [a]Unpaired t-test with Welch's correction, [b]Mann-Whitney test. Middle-aged: n = 6 - 7 per group, Young adult: n = 4 per group. SHAM - sham operation, OVX - bilateral ovariectomy.

Supplementary Table 3, Additional file 3. Results of the two-way ANOVA analysis of the daily distance covered during 24 hours of six weeks of voluntary wheel running.

	**Time**		**Operation**		**Interaction (time x operation)**	
**Distance (km)**	F (DFn, D Fd)	p-value	F (DFn, DFd)	p-value	F (DFn, DFd)	p-value
**Middle-aged female**	F (2.867, 217.9) = 113.7	< 0.0001	F (1, 76) = 25.01	< 0.0001	F (23, 1748) = 20.61	< 0.0001
**Young adult females**	F (3.152, 145.0) = 149.7	< 0.0001	F (1, 46) = 66.31	< 0.0001	F (23, 1058) = 41.98	< 0.0001

Middle-aged: n = 6 - 7 per group, Young adult: n = 4 per group. DFn - numerator degrees of freedom, DFd - the denominator degrees of freedom.

Supplementary Table 4, Additional file 3. Results of the two-way ANOVA analysis of middle-aged mice's frontal cortex protein expression level seven weeks post-operation.

	**Middle-aged females**					
	**Intervention (VWR/MF)**		**Operation**		**Interaction (intervention x operation)**	
	F (DFn, DFd)	p-value	F (DFn, DFd)	p-value	F (DFn, DFd)	p-value
**NLRP3**	F (2, 21) = 3.56	0.0468	F (1, 21) = 1.50	0.2346	F (2, 21) = 8.39	0.0021
**Pro-caspase 1**	F (2, 21) = 0.13	0.8807	F (1, 21) = 0.05	0.8171	F (2, 21) = 0.26	0.7734
**ASC**	F (2, 21) = 4.02	0.0334	F (1, 21) = 0.29	0.5964	F (2, 21) = 11.14	0.0005
**Pro-IL-1β**	F (2, 21) = 3.93	0.0356	F (1, 21) = 0.01	0.9367	F (2, 21) = 0.31	0.7398
**Pro-IL-18**	F (2, 21) = 0.17	0.8417	F (1, 21) = 0.21	0.6508	F (2, 21) = 2.47	0.1090
**TLR4**	F (2, 21) = 0.82	0.4523	F (1, 21) = 6.46	0.0190	F (2, 21) = 0.88	0.4312
**NF-κB p65**	F (2, 21) = 0.16	0.8518	F (1, 21) = 0.14	0.7152	F (2, 21) = 0.03	0.9751
**Phospho-NF-κB p65 (p-p65)**	F (2, 21) = 0.28	0.7551	F (1, 21) = 0.89	0.3553	F (2, 21) = 0.02	0.9806
**p-p65/p65 ratio**	F (2, 21) = 0.83	0.4512	F (1, 21) = 0.43	0.5185	F (2, 21) = 0.41	0.6749

n = 4 - 6 per group. DFn - numerator degrees of freedom, DFd - the denominator degrees of freedom, VWR - voluntary wheel running, MF – metformin treatment

Supplementary Table 5, Additional file 3. Results of the two-way ANOVA analysis of young adult mice's frontal cortex protein expression level seven weeks post-operation.

	Young adult females					
	Intervention (VWR/MF)		Operation		Interaction (intervention x operation)	
	F (DFn, DFd)	p-value	F (DFn, DFd)	p-value	F (DFn, DFd)	p-value
**NLRP3**	F (2, 18) = 5.92	0.0106	F (1, 18) = 0.47	0.5001	F (2, 18) = 3.28	0.0612
**Pro-caspase 1**	F (2, 18) = 1.75	0.2019	F (1, 18) = 0.08	0.7741	F (2, 18) = 7.03	0.0055
**ASC**	F (2, 18) = 0.98	0.3953	F (1, 18) = 1.29	0.2702	F (2, 18) = 2.37	0.1220
**Pro-IL-1β**	F (2, 18) = 1.09	0.3579	F (1, 18) = 10.64	0.0043	F (2, 18) = 0.29	0.7495
**Pro-IL-18**	F (2, 18) = 0.69	0.5151	F (1, 18) = 4.51	0.0478	F (2, 18) = 0.64	0.5364
**TLR4**	F (2, 18) = 16.19	<0.0001	F (1, 18) = 3.32	0.0850	F (2, 18) = 0.64	0.5365
**NF-κB p65**	F (2, 18) = 17.04	<0.0001	F (1, 18) = 0.06	0.8150	F (2, 18) = 5.58	0.0131
**Phospho-NF-κB p65 (p-p65)**	F (2, 18) = 0.63	0.5427	F (1, 18) = 5.08	0.0369	F (2, 18) = 4.62	0.0241
**p-p65/p65 ratio**	F (2, 18) = 5.45	0.0141	F (1, 18) = 0.89	0.3567	F (2, 18) = 10.56	0.0009

n = 4 per group. DFn - numerator degrees of freedom, DFd - the denominator degrees of freedom, VWR - voluntary wheel running, MF – metformin treatment

Supplementary Table 6, Additional file 3. Results of the two-way ANOVA analysis of middle-aged mice's frontal cortex gene expression level seven weeks post-operation.

	**Middle-aged females**					
	**Intervention (VWR/MF)**		**Operation**		**Interaction (intervention x operation)**	
	F (DFn, DFd)	p-value	F (DFn, DFd)	p-value	F (DFn, DFd)	p-value
Nlrp3	F (2, 40) = 0.95	0.3946	F (1, 40) = 3.95	0.0536	F (2, 40) = 0.47	0.6266
Casp1	F (2, 40) = 0.94	0.3989	F (1, 40) = 2.82	0.1008	F (2, 40) = 1.51	0.2334
Il-1b	F (2, 40) = 1.25	0.2975	F (1, 40) = 6.40	0.0155	F (2, 40) = 1.30	0.2837
Il-18	F (2, 38) = 3.79	0.0317	F (1, 38) = 0.26	0.6162	F (2, 38) = 0.03	0.9748
Tlr4	F (2, 40) = 2.25	0.1183	F (1, 40) = 0.57	0.4554	F (2, 40) = 0.29	0.7535
Rela	F (2, 40) = 2.51	0.0943	F (1, 40) = 4.12	0.0492	F (2, 40) = 0.12	0.8854
Relb	F (2, 40) = 3.72	0.0330	F (1, 40) = 5.73	0.0214	F (2, 40) = 1.24	0.3009
Nfkbia	F (2, 40) = 3.01	0.0610	F (1, 40) = 1.87	0.1791	F (2, 40) = 2.08	0.1387
Nfkbib	F (2, 40) = 2.48	0.0966	F (1, 40) = 0.07	0.7904	F (2, 40) = 0.26	0.7757
Ikbkb	F (2, 40) = 4.86	0.0129	F (1, 40) = 1.56	0.2196	F (2, 40) = 0.34	0.7160

n = 6 - 9 per group. DFn - numerator degrees of freedom, DFd - the denominator degrees of freedom, VWR - voluntary wheel running, MF – metformin treatment

Supplementary Table 7, Additional file 3. Results of the two-way ANOVA analysis of young adult mice's frontal cortex gene expression level seven weeks post-operation.

	Young adult females					
	**Intervention (VWR/MF)**		**Operation**		**Interaction (intervention x operation)**	
	F (DFn, DFd)	p-value	F (DFn, DFd)	p-value	F (DFn, DFd)	p-value
Nlrp3	F (2, 21) = 2.38	0.1174	F (1, 21) = 1.86	0.1870	F (2, 21) = 0.85	0.4428
Casp1	F (2, 21) = 1.757	0.1970	F (1, 21) = 0.03	0.8586	F (2, 21) = 0.16	0.8518
Il-1b	F (2, 21) = 4.27	0.0278	F (1, 21) = 0.01	0.9663	F (2, 21) = 0.27	0.7632
Il-18	F (2, 21) = 1.788	0.1918	F (1, 21) = 0.34	0.5674	F (2, 21) = 0.51	0.6113
Tlr4	F (2, 21) = 0.77	0.4756	F (1, 21) = 0.02	0.8960	F (2, 21) = 0.58	0.5684
Rela	F (2, 21) = 0.27	0.7665	F (1, 21) = 0.95	0.3408	F (2, 21) = 0.04	0.9593
Relb	F (2, 21) = 2.58	0.0998	F (1, 21) = 0.44	0.5138	F (2, 21) = 0.34	0.7147
Nfkbia	F (2, 21) = 3.92	0.0357	F (1, 21) = 1.28	0.2714	F (2, 21) = 0.60	0.5603
Nfkbib	F (2, 21) = 0.74	0.4904	F (1, 21) = 4.41	0.0482	F (2, 21) = 0.15	0.8607
Ikbkb	F (2, 21) = 0.25	0.7836	F (1, 21) = 0.09	0.7652	F (2, 21) = 0.05	0.9504

n = 4 - 5 per group. DFn - numerator degrees of freedom, DFd - the denominator degrees of freedom, VWR - voluntary wheel running, MF – metformin treatment

Supplementary Table 8, Additional file 3. Results of the two-way ANOVA analysis of middle-aged and young adult mice's serum IL-1β, IL-18, and TNF-α seven weeks post-operation.

		**Intervention (VWR/MF)**		**Operation**		**Interaction (intervention x operation)**	
		F (DFn, DFd)	p-value	F (DFn, DFd)	p-value	F (DFn, DFd)	p-value
**Middle-aged females**	IL-1β	F (2, 20) = 0.65	0.5319	F (1, 20) = 0.31	0.5842	F (2, 20) = 1.56	0.2354
	IL-18	F (2, 40) = 5.32	0.0089	F (1, 40) = 0.39	0.5357	F (2, 40) = 1.24	0.3016
	TNF-α	F (2, 39) = 2.65	0.0836	F (1, 39) = 7.22	0.0105	F (2, 39) = 1.81	0.1765
**Young adult females**	IL-1β	F (2, 18) = 0.28	0.7585	F (1, 18) = 5.63	0.0290	F (2, 18) = 1.27	0.3045
	IL-18	F (2, 21) = 0.55	0.5823	F (1, 21) = 0.08	0.7854	F (2, 21) = 0.23	0.7966
	TNF-α	F (2, 20) = 2.72	0.0906	F (1, 20) = 2.61	0.1223	F (2, 20) = 0.59	0.5638

Middle-aged: n = 6 - 7 per group, Adult: n = 4 per group. DFn - numerator degrees of freedom, DFd - the denominator degrees of freedom, VWR - voluntary wheel running, MF – metformin treatment
