# Supplementary material for: Age-dependent neuroinflammation response to voluntary wheel running and Metformin treatment in the frontal cortex of ovariectomized female mice
Source: Sci Rep. 2025 Jul 21;15:26382. doi: 10.1038/s41598-025-10014-0 (PMC12280059; doi:10.1038/s41598-025-10014-0)
Supplement: Supplementary file 3 — Supplementary Material 3 [file 41598_2025_10014_MOESM3_ESM.pdf]

Additional File 2

Age-dependent neuroinflammation response to voluntary wheel running and metformin treatment in the frontal cortex of ovariectomized female mice

Konstancja Grabowska[1,2*], Mateusz Grabowski[2], Julia Morys[2], Edyta Olakowska[1], Andrzej Małecki[2], Jarosław J Barski[1], and Marta Nowacka-Chmielewska[2]

[1]Department of Physiology, Faculty of Medical Sciences in Katowice, Medical University of Silesia, Poland

[2]Laboratory of Molecular Biology, Institute of Physiotherapy and Health Sciences, Academy of Physical Education, Katowice, Poland

* Corresponding author: konstancja.grabowska@sum.edu.pl

keywords: neuroinflammation, NLRP3 inflammasome, voluntary wheel running, metformin, ovariectomy, physical activity

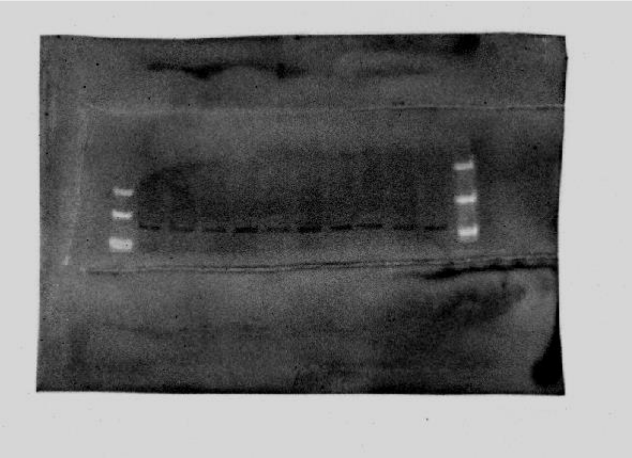

Fig. 1 Chemiluminescence visualization of NLRP3 bands on western blot membrane from the experiment 1.

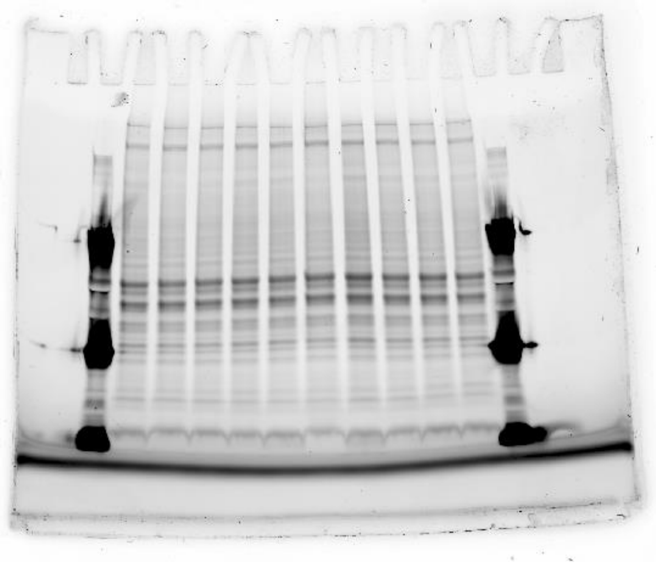

Fig. 2 Visualization of total protein on polyacrylamide pre-cast gel (Bio-Rad) after electrophoresis under UV light as loading control to NLRP3 from the experiment 1.

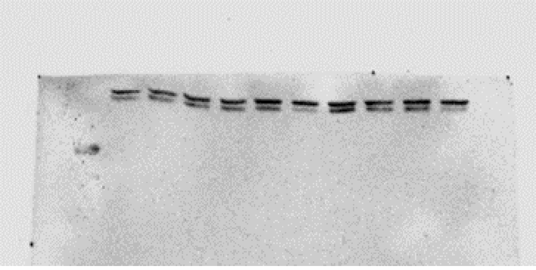

Fig. 3 Chemiluminescence visualization of pro-caspase 1 bands on western blot membrane from the experiment 1.

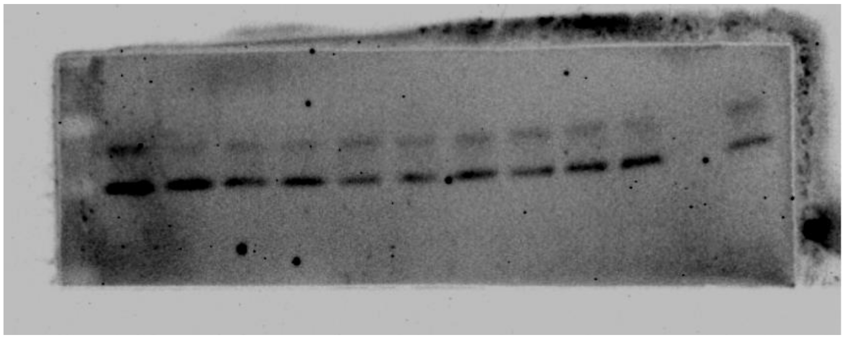

Fig. 4 Chemiluminescence visualization of ASC bands on western blot membrane from the experiment 1.

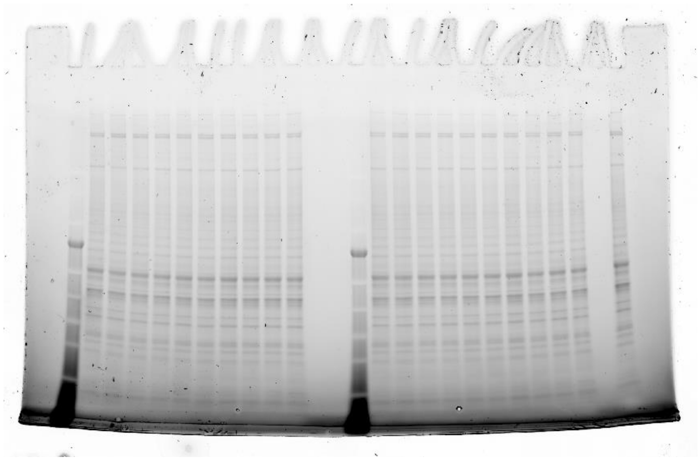

Fig. 5 Visualization of total protein on polyacrylamide pre-cast gel (Bio-Rad) after electrophoresis under UV light as loading control to pro-caspase-1 (left side) and ASC (right side) from the experiment 1.

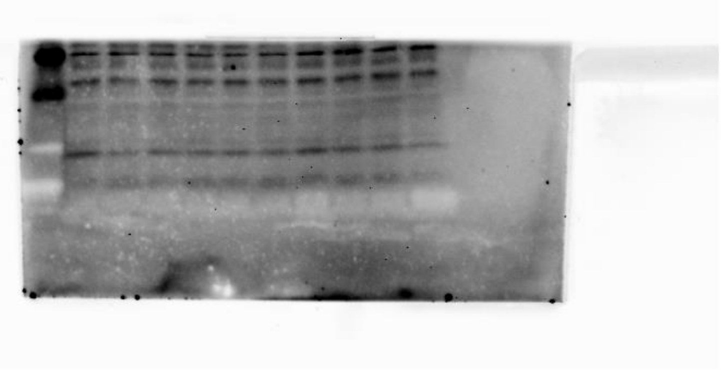

Fig. 6 Chemiluminescence visualization of pro-IL-1β bands on western blot membrane from the experiment 1.

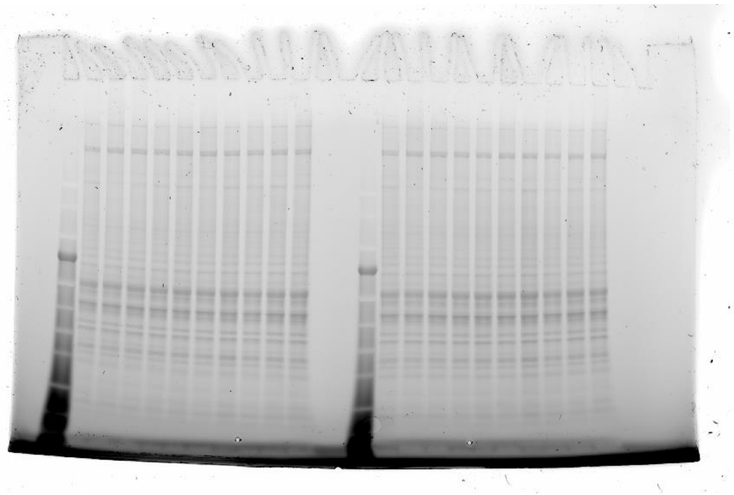

Fig. 7 Visualization of total protein on polyacrylamide pre-cast gel (Bio-Rad) after electrophoresis under UV light as loading control to pro-IL-1β and TLR4 (right side) from the experiment 1.

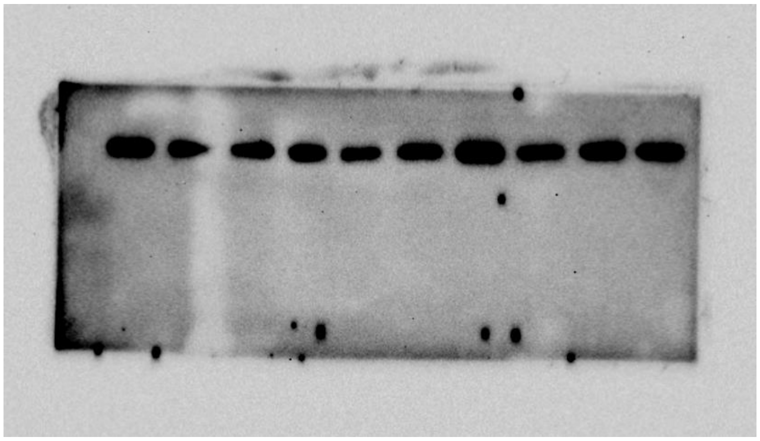

Fig. 8 Chemiluminescence visualization of pro-IL-18 bands on western blot membrane from the experiment 1.

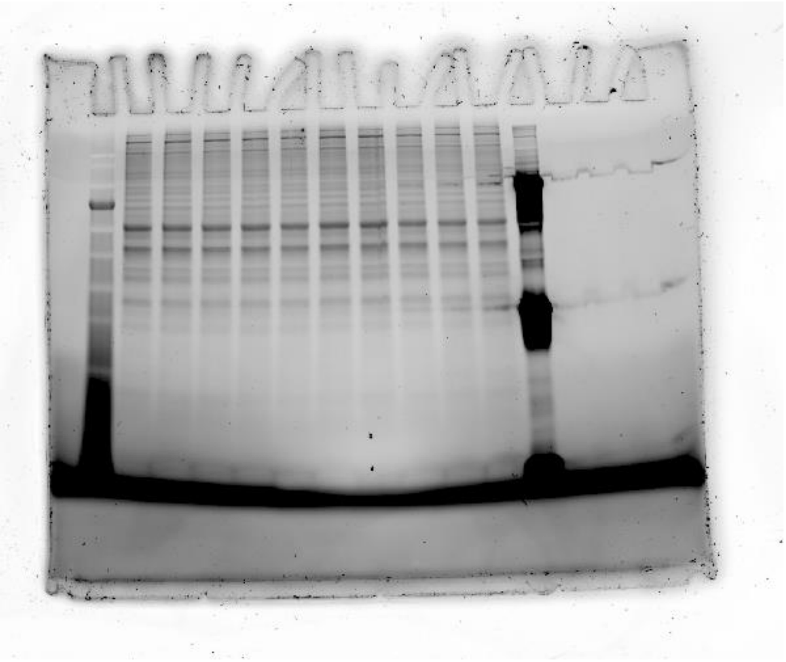

Fig. 9 Visualization of total protein on polyacrylamide pre-cast gel (Bio-Rad) after electrophoresis under UV light as loading control to pro-IL-18 and NF-κB p65 from the experiment 1.

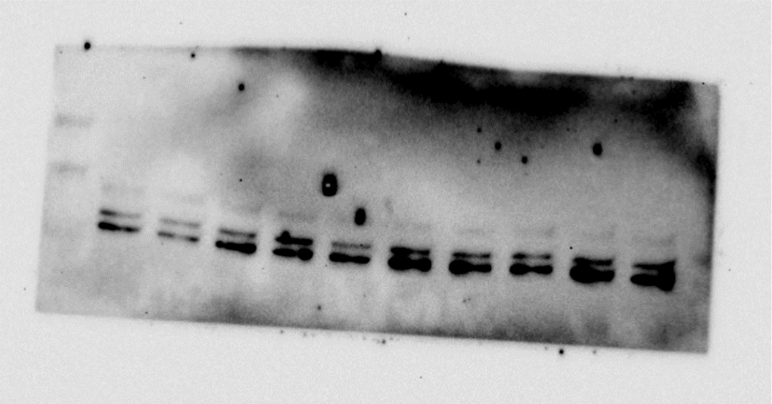

Fig. 10 Chemiluminescence visualization of TLR4 bands on western blot membrane from the experiment 1.

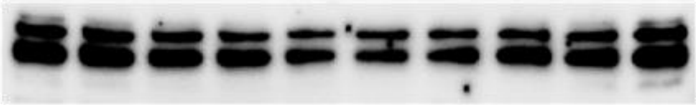

Fig. 11 Chemiluminescence visualization of NF-κB p65 bands on western blot membrane from the experiment 1.

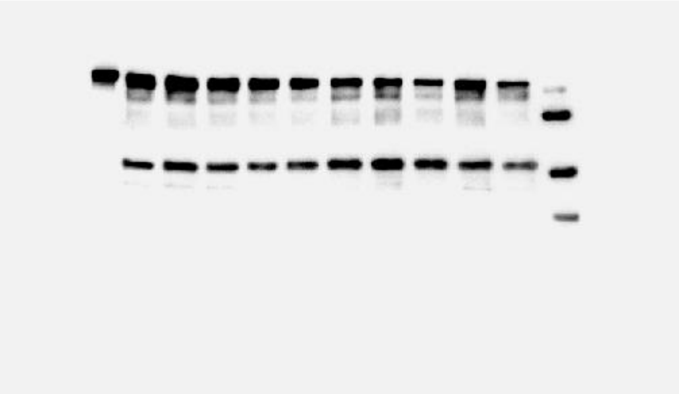

Fig. 12 Chemiluminescence visualization of phosphop-NF-κB p65 bands on western blot membrane from the experiment 1.

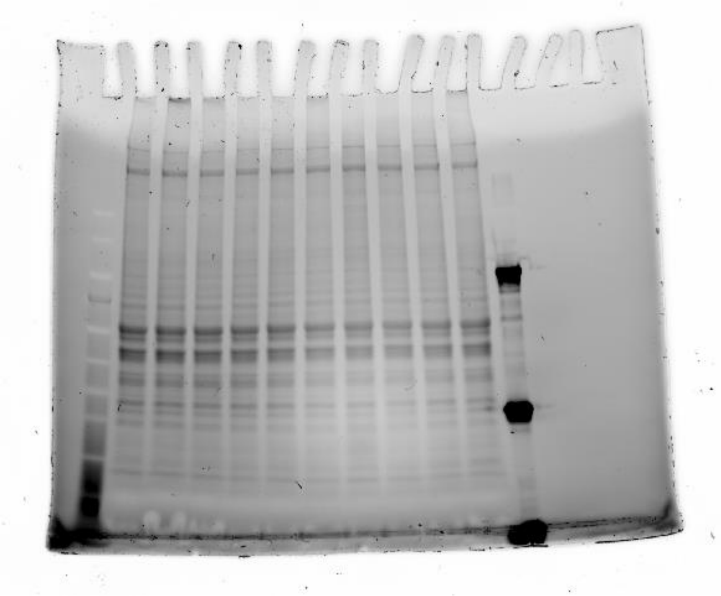

Fig. 13 Visualization of total protein on polyacrylamide pre-cast gel (Bio-Rad) after electrophoresis under UV light as loading control to phospho-NF-κB p65 from the experiment 1.

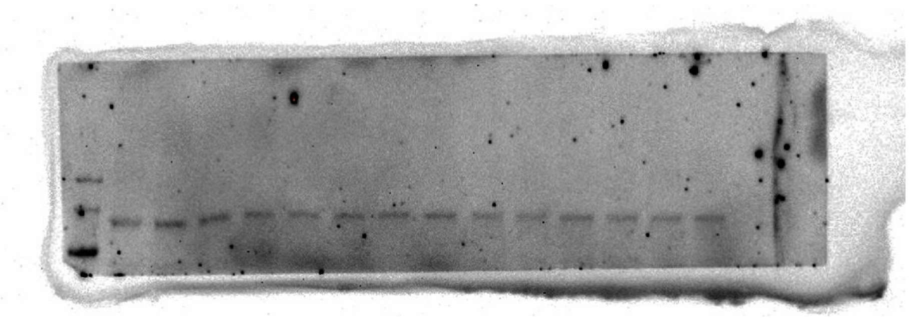

Fig. 14 Chemiluminescence visualization of NLRP3 bands on western blot membrane from the experiment 2 A.

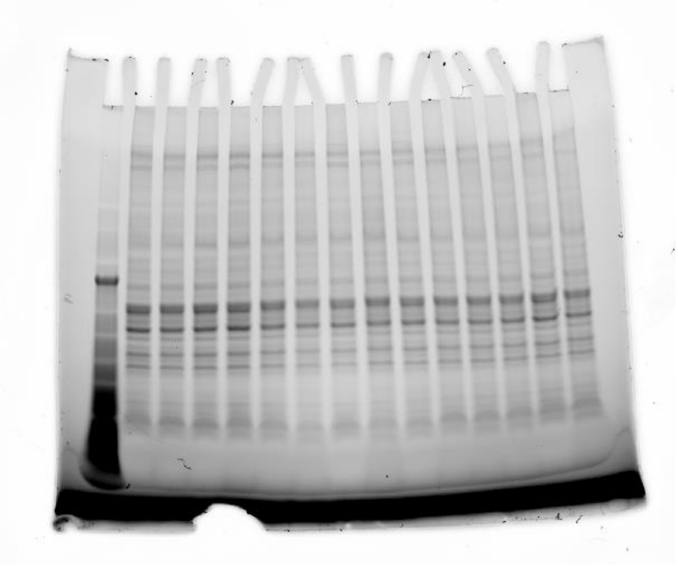

Fig. 15 Visualization of total protein on polyacrylamide pre-cast gel (Bio-Rad) after electrophoresis under UV light as loading control to NLRP3 from the experiment 2 A.

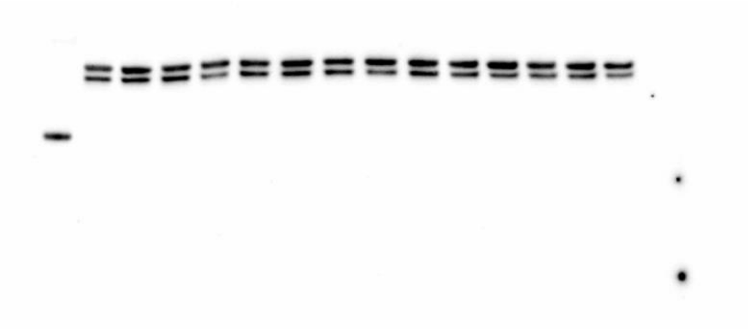

Fig. 16 Chemiluminescence visualization of pro-caspase 1 bands on western blot membrane from the experiment 2 A.

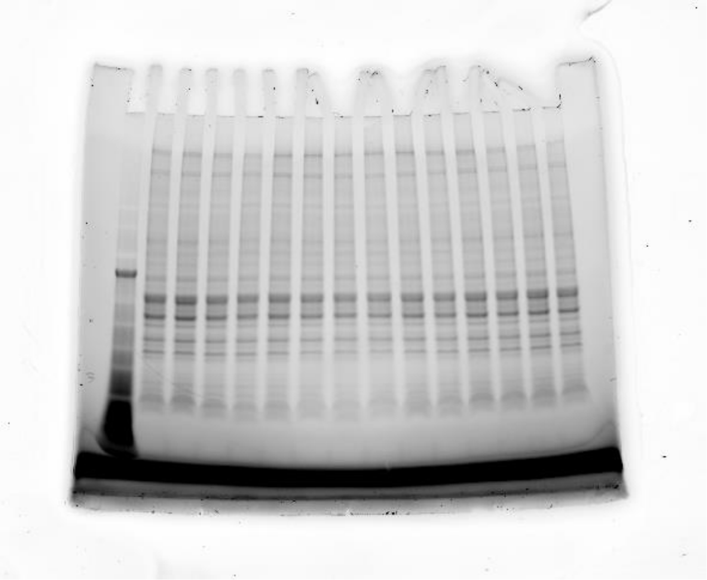

Fig. 17 Visualization of total protein on polyacrylamide pre-cast gel (Bio-Rad) after electrophoresis under UV light as loading control to pro-caspase 1 from the experiment 2 A.

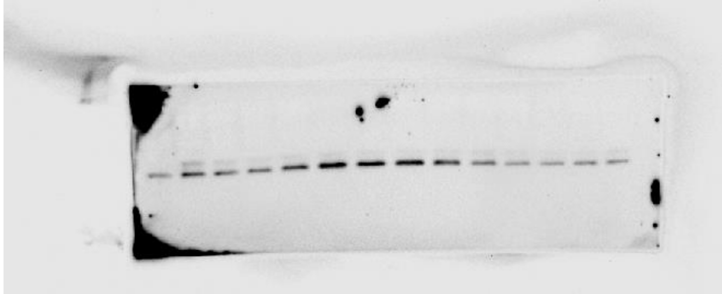

Fig. 18 Chemiluminescence visualization of ASC bands on western blot membrane from the experiment 2 A.

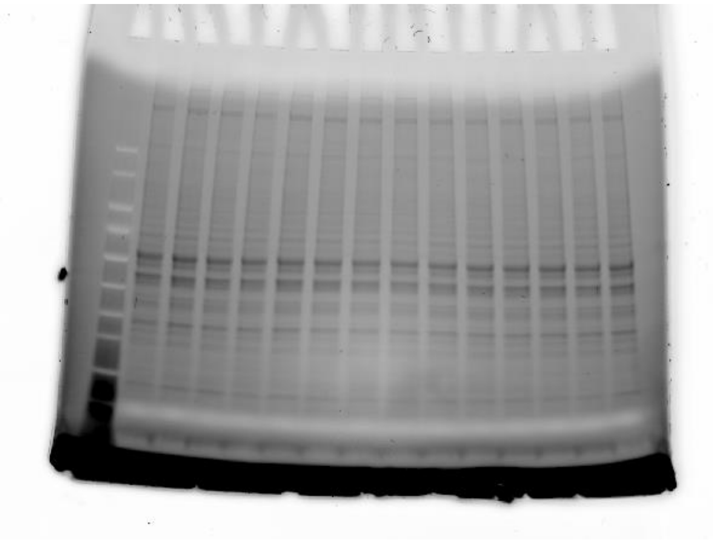

Fig. 19 Visualization of total protein on polyacrylamide pre-cast gel (Bio-Rad) after electrophoresis under UV light as loading control to ASC from the experiment 2 A.

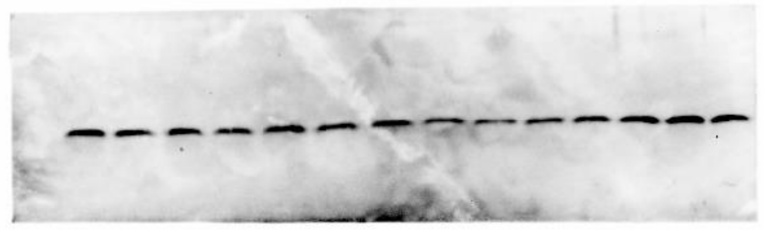

Fig. 20 Chemiluminescence visualization of pro-IL-1β bands on western blot membrane from the experiment 2 A.

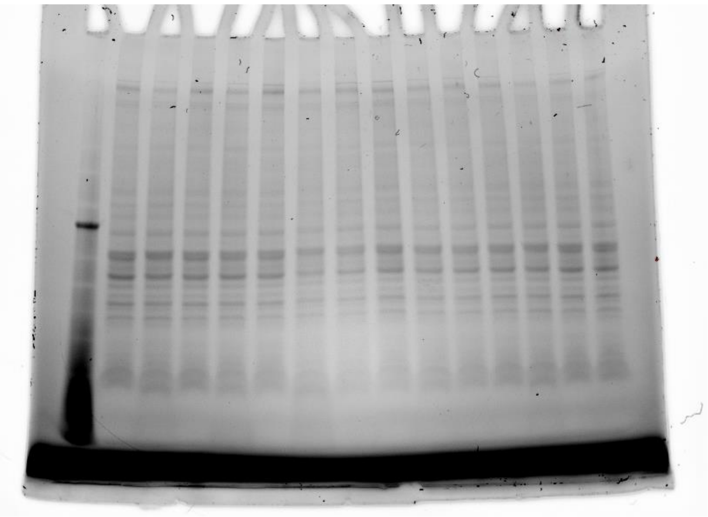

Fig. 21 Visualization of total protein on polyacrylamide pre-cast gel (Bio-Rad) after electrophoresis under UV light as loading control to pro-IL-1β from the experiment 2 A.

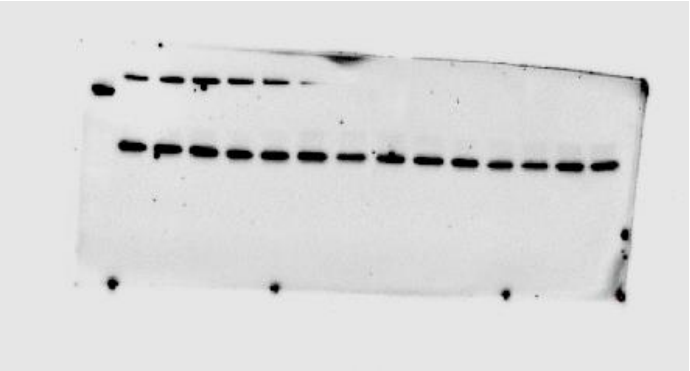

Fig. 22 Chemiluminescence visualization of pro-IL-18 bands on western blot membrane from the experiment 2 A.

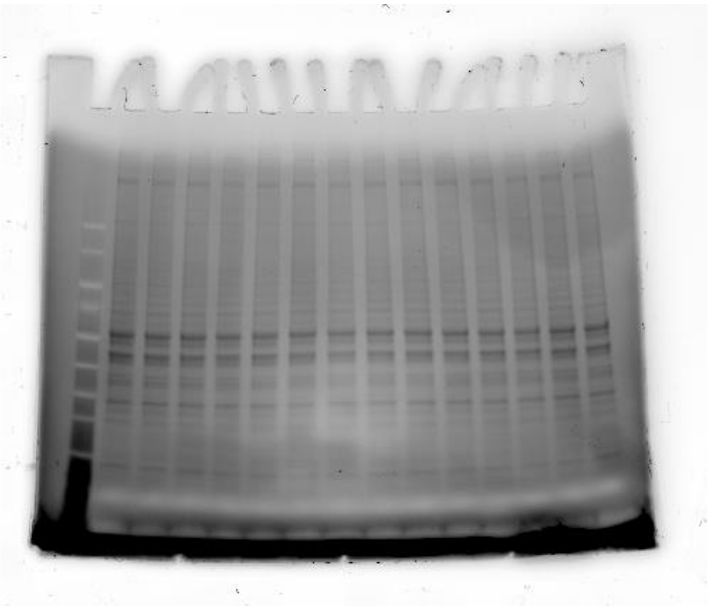

Fig. 23 Visualization of total protein on polyacrylamide pre-cast gel (Bio-Rad) after electrophoresis under UV light as loading control to pro-IL-18 from the experiment 2 A.

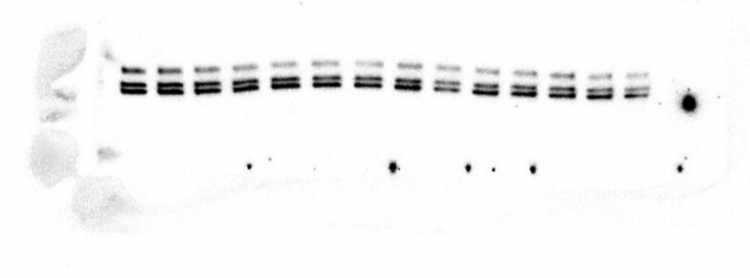

Fig. 24 Chemiluminescence visualization of TLR4 bands on western blot membrane from the experiment 2 A.

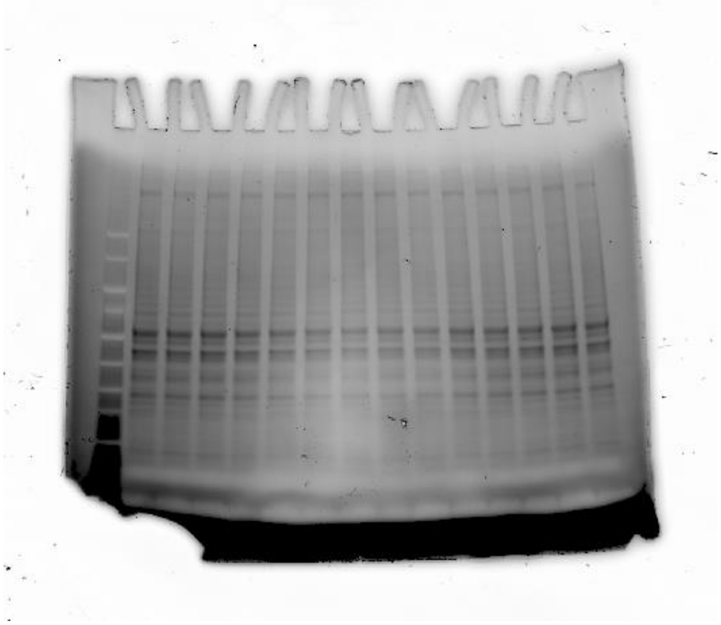

Fig. 25 Visualization of total protein on polyacrylamide pre-cast gel (Bio-Rad) after electrophoresis under UV light as loading control to TLR4 from the experiment 2 A.

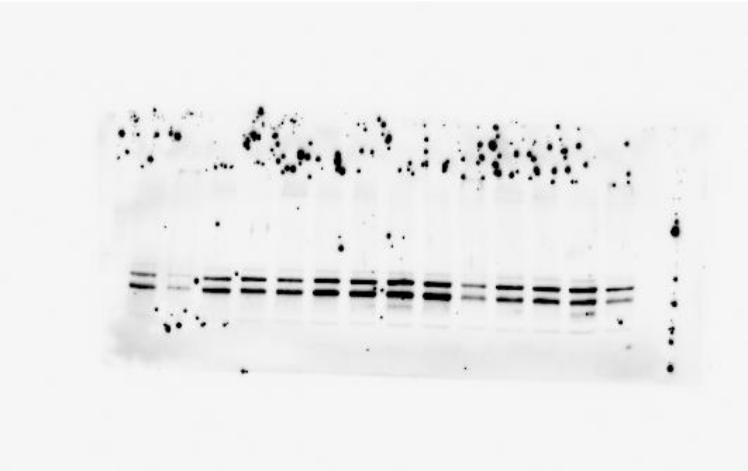

Fig. 26 Chemiluminescence visualization of NF-κB p65 bands on western blot membrane from the experiment 2 A.

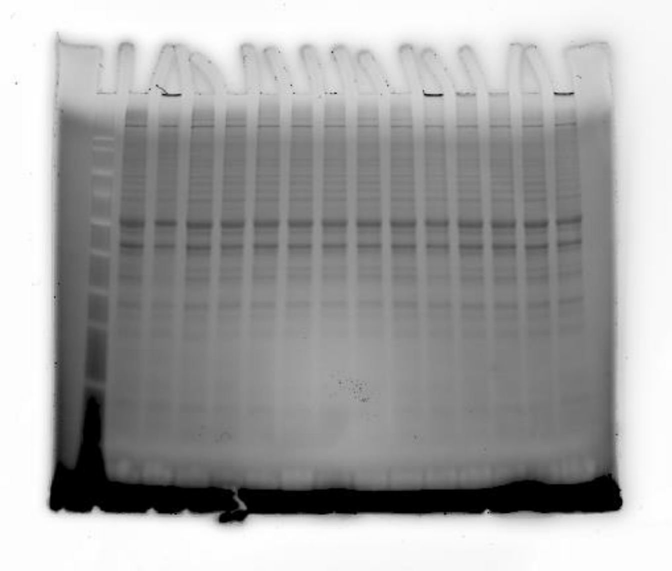

Fig. 27 Visualization of total protein on polyacrylamide pre-cast gel (Bio-Rad) after electrophoresis under UV light as loading control to NF-κB p65 from the experiment 2 A.

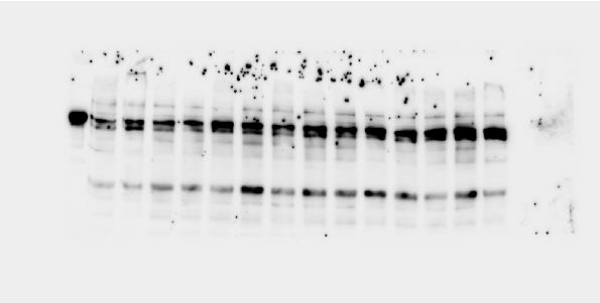

Fig. 28 Chemiluminescence visualization of phosphop-NF-κB p65 bands on western blot membrane from the experiment 2 A.

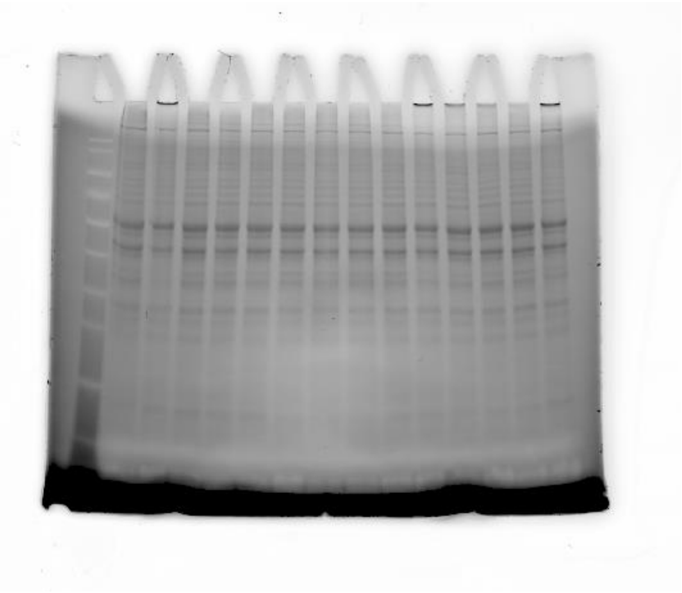

Fig. 29 Visualization of total protein on polyacrylamide pre-cast gel (Bio-Rad) after electrophoresis under UV light as loading control to phosphop-NF-κB p65 from the experiment 2 A.

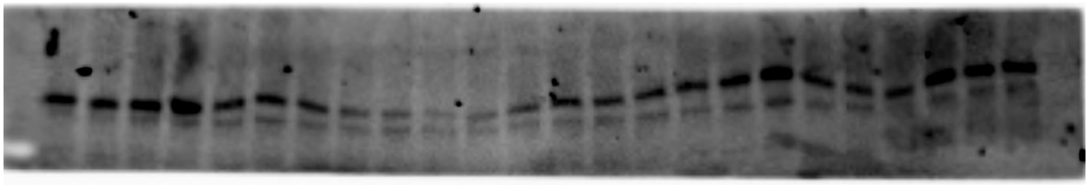

Fig. 30 Chemiluminescence visualization of NLRP3 bands on western blot membrane from the experiment 2 B.

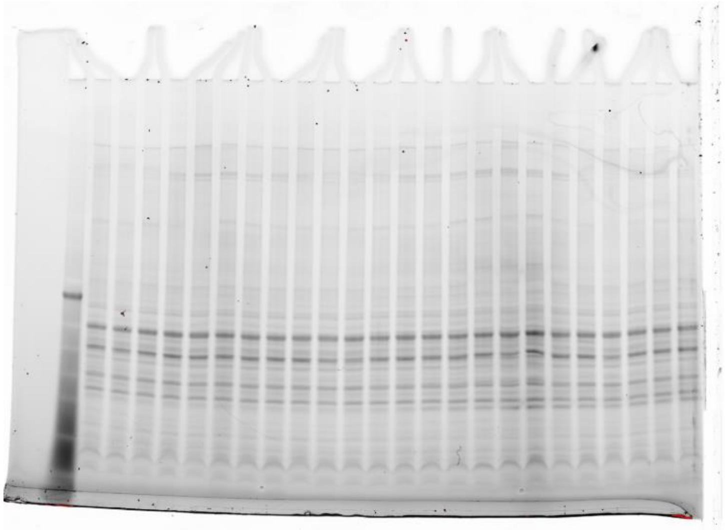

Fig. 31 Visualization of total protein on polyacrylamide pre-cast gel (Bio-Rad) after electrophoresis under UV light as loading control to NLRP3 from the experiment 2 B.

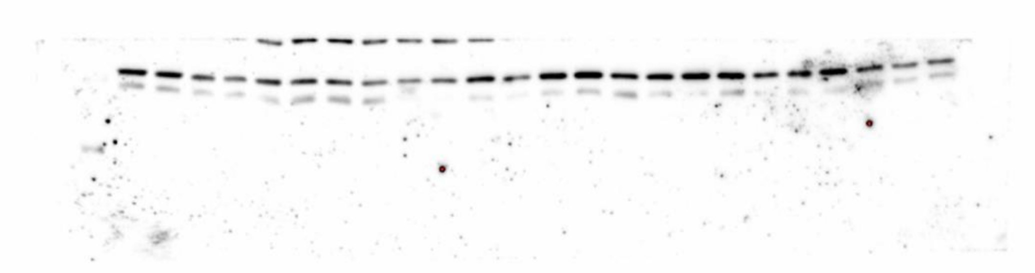

Fig. 32 Chemiluminescence visualization of pro-caspase 1 bands on western blot membrane from the experiment 2 B.

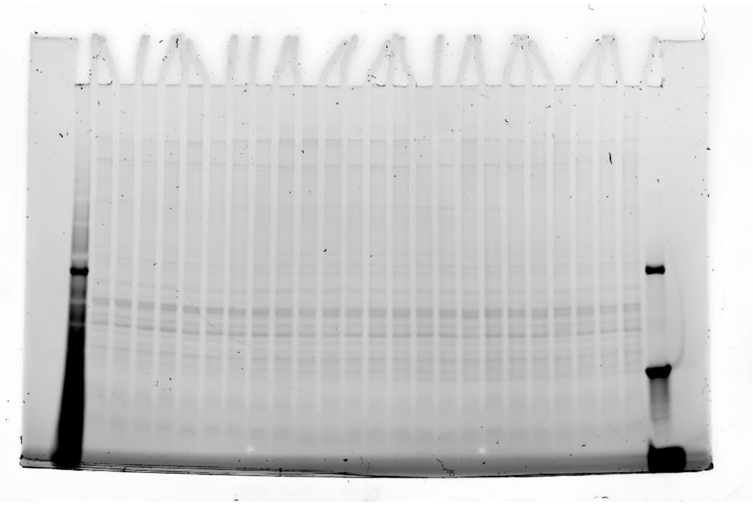

Fig. 33 Visualization of total protein on polyacrylamide pre-cast gel (Bio-Rad) after electrophoresis under UV light as loading control to pro-caspase 1 and TLR4 from the experiment 2 B.

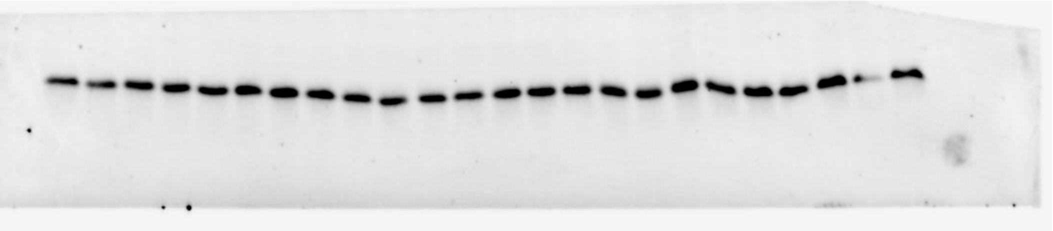

Fig. 34 Chemiluminescence visualization of ASC bands on western blot membrane from the experiment 2 B.

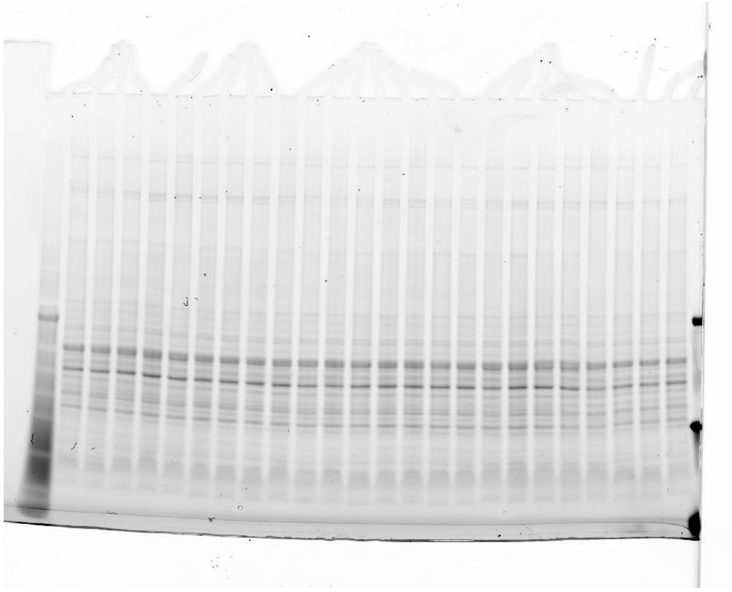

Fig. 35 Visualization of total protein on polyacrylamide pre-cast gel (Bio-Rad) after electrophoresis under UV light as loading control to ASC and phosphop-NF-κB p65 from the experiment 2 B.

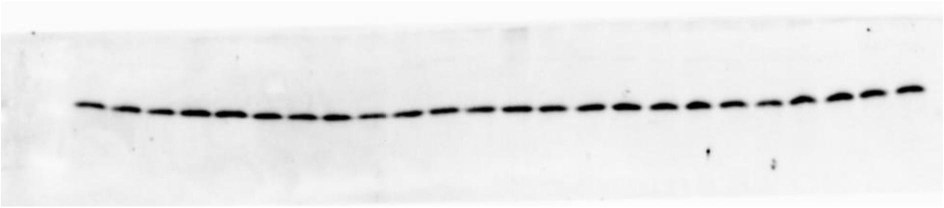

Fig. 36 Chemiluminescence visualization of pro-IL-1β bands on western blot membrane from the experiment 2 B.

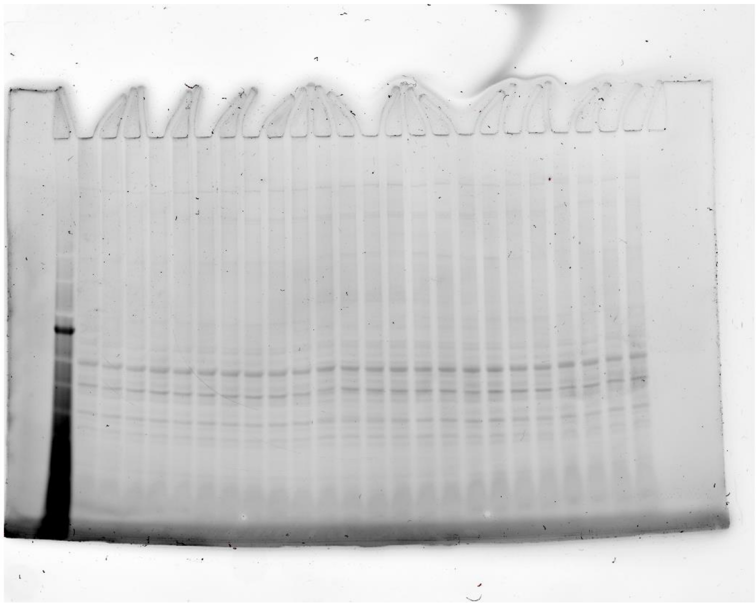

Fig. 37 Visualization of total protein on polyacrylamide pre-cast gel (Bio-Rad) after electrophoresis under UV light as loading control to pro-IL-1β from the experiment 2 B.

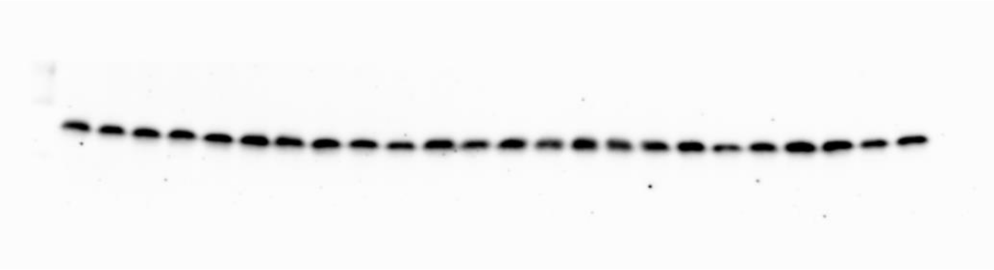

Fig. 38 Chemiluminescence visualization of pro-IL-18 bands on western blot membrane from the experiment 2 B.

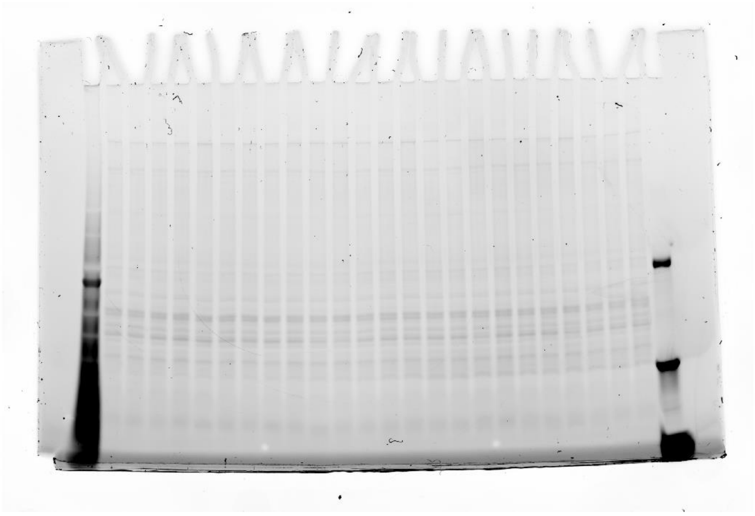

Fig. 39 Visualization of total protein on polyacrylamide pre-cast gel (Bio-Rad) after electrophoresis under UV light as loading control to pro-IL-18 from the experiment 2 B.

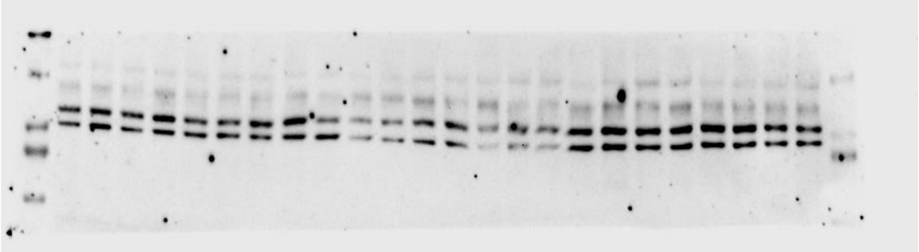

Fig. 40 Chemiluminescence visualization of TLR4 bands on western blot membrane from the experiment 2 B.

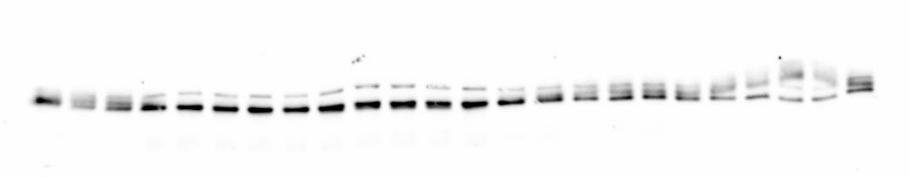

Fig. 41 Chemiluminescence visualization of NF-κB p65 bands on western blot membrane from the experiment 2B.

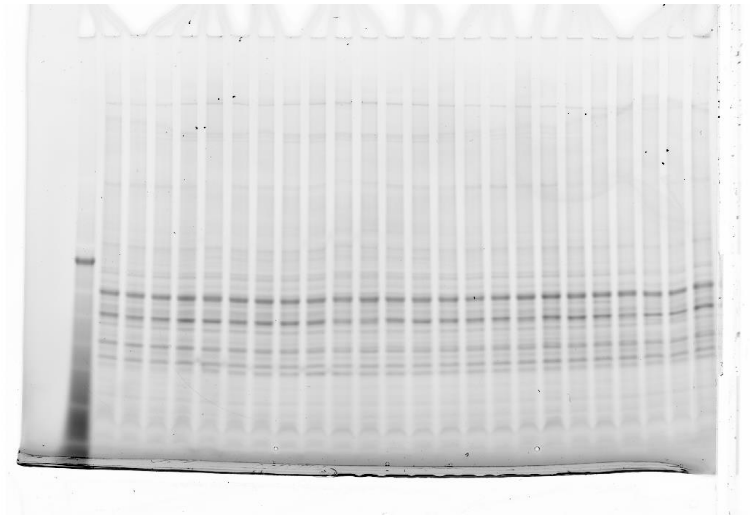

Fig. 42 Visualization of total protein on polyacrylamide pre-cast gel (Bio-Rad) after electrophoresis under UV light as loading control to NF-κB p65 from the experiment 2 B.

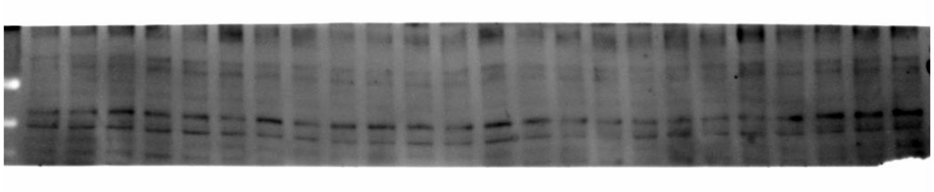

Fig. 43 Chemiluminescence visualization of phosphop-NF-κB p65 bands on western blot membrane from the experiment 2 B.
